# miR-34 activity is modulated through 5′-end phosphorylation in response to DNA damage

**DOI:** 10.1038/ncomms10954

**Published:** 2016-03-21

**Authors:** David W. Salzman, Kotoka Nakamura, Sunitha Nallur, Michelle T. Dookwah, Chanatip Metheetrairut, Frank J. Slack, Joanne B. Weidhaas

**Affiliations:** 1 Department of Therapeutic Radiology, Yale School of Medicine, 15 York Street, New Haven, Connecticut 06502, USA; 2 Department of Radiation Oncology, David Geffen School of Medicine, Los Angeles, California 90024, USA; 3 Department of Molecular, Cellular and Developmental Biology, Yale University, PO Box 208103, New Haven, Connecticut 06502, USA; 4 Department of Pathology, Beth Israel Deaconess Medical Center, Institute for RNA Medicine, Harvard Medical School, 330 Brookline Avenue, Boston, Massachusetts 02215, USA

## Abstract

MicroRNA (miRNA) expression is tightly regulated by several mechanisms, including transcription and cleavage of the miRNA precursor RNAs, to generate a mature miRNA, which is thought to be directly correlated with activity. MiR-34 is a tumour-suppressor miRNA important in cell survival, that is transcriptionally upregulated by p53 in response to DNA damage. Here, we show for the first time that there is a pool of mature miR-34 in cells that lacks a 5′-phosphate and is inactive. Following exposure to a DNA-damaging stimulus, the inactive pool of miR-34 is rapidly activated through 5′-end phosphorylation in an ATM- and Clp1-dependent manner, enabling loading into Ago2. Importantly, this mechanism of miR-34 activation occurs faster than, and independently of, *de novo* p53-mediated transcription and processing. Our study reveals a novel mechanism of rapid miRNA activation in response to environmental stimuli occurring at the mature miRNA level.

## Abstract (web summary)

MiR-34 is a tumour suppressor microRNA known to be upregulated by p53 after DNA damage and plays a critical role in cell cycle arrest and apoptosis. Here the authors show the cell maintains an inactive pool of miR-34 which is rapidly activated after damage via ATM-dependent phosphorylation.

## Graphical abstract

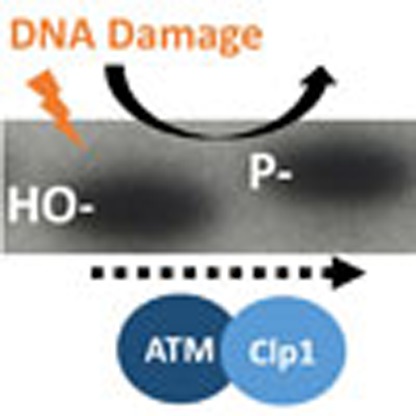

MicroRNA (miRNA) expression is regulated by several mechanisms, including transcription and cleavage of the miRNA precursors by Drosha and Dicer to generate a mature miRNA^1^,^2^. It is widely believed that the expression of the mature miRNA is directly correlated with gene silencing activity. MiR-34 is a tumour-suppressor miRNA, important in cell cycle and cell survival control, which has entered clinical trials as a cancer therapeutic^3^. In mammals, miR-34 is transcriptionally upregulated by p53 in response to DNA damage^4^,^5^,^6^, and has been shown to play a critical role in determining cell fate after such damage by targeting a number of genes involved in cell cycle arrest and apoptosis^7^.

Systematic deletion of miRNA genes in *Caenorhabditis elegans*^8^ and mice^9^ indicates that the majority of miRNAs are not essential for development and biological processes, suggesting that the purpose of many miRNAs may be to function in the cellular stress response, and that their biological function can only be elucidated in a context-specific manner^10^. For example, *C. elegans* harbouring a deletion of *mir-34* displays no abnormal morphological, developmental or biological phenotypes under normal conditions. However, these animals are hypersensitivite to radiation-induced DNA damage^11^ and exhibit developmental defects under stress conditions^12^. In mammals, miR-34 is also critical in the DNA damage response, and its expression is transcriptionally regulated by p53 in response to numerous forms of DNA damage^4^,^5^,^6^.

Here, we show for the first time that in the absence of DNA damage there is a pool of mature, inactive miR-34 in cells, which lacks a 5′-phosphate and is not loaded into Ago2. When cells are exposed to ionizing radiation (IR), this pool is rapidly activated through 5′-end phosphorylation, which is ataxia telangiectasia mutated (ATM)-dependent, involves Clp1, and results in Ago2 loading. Importantly, ATM-dependent 5′-end phosphorylation occurs faster than, and independently of, *de novo* p53-mediated transcription and processing. Our study reveals a novel mechanism of rapid activation of miRNA activity in response to an environmental stimuli, DNA damage, which occurs at the level of the mature miRNA.

## Results

### Evidence for a pool of inactive mature miR-34

**Figure 1:**
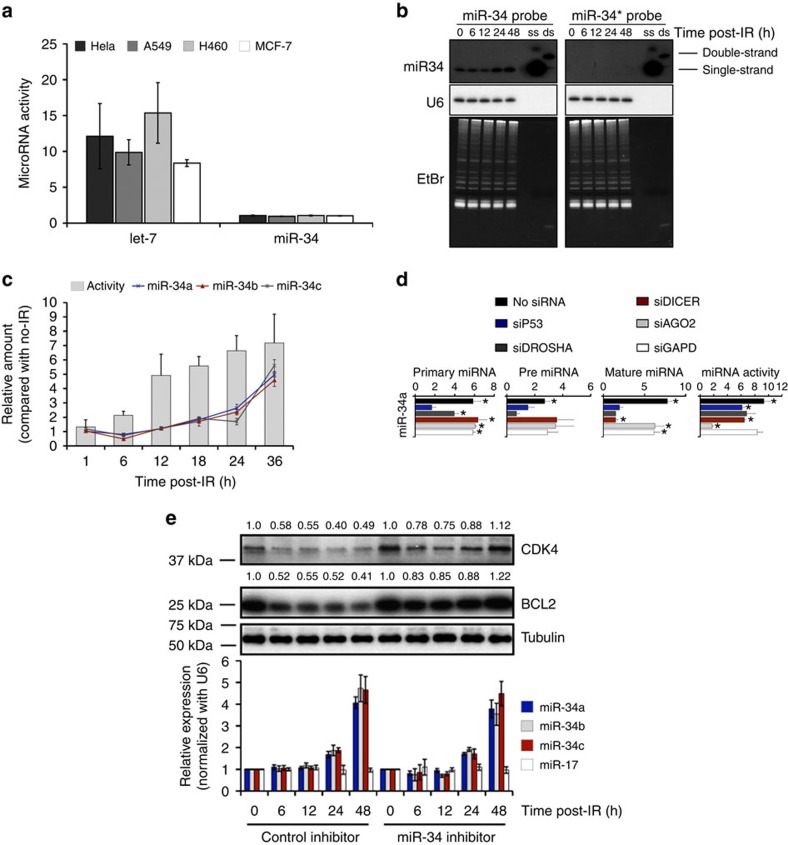
DNA damage activates a pool of existing, mature miR-34 that leads to strong gene repression. (**a**) miR-34 and *let-7* activity in cells 16 h after transfection with dual-luciferase psi-miR-34 (WT or MT) and psi-let-7 (WT or MT) reporters. Renilla was normalized to Firefly and miRNA activity was expressed as the fold-suppression of MT/WT. Graphed is the average±s.d. of three experiments in triplicate. (**b**) Northern blot of miR-34 and miR-34* from A549 cells exposed to 6 Gy of IR. U6 and ethidium bromide-stained gels were used as a loading control. (**c**) A549 cells expressing psi-miR-34 (WT or MT) reporters were lysed at the indicated time after exposure to 4 Gy. Lysates were assayed for dual-luciferase and mature miR-34a/b/c expression by RT–qPCR. Renilla was normalized to Firefly and was expressed as the fold-suppression of MT/WT. MiR-34 expression was normalized to U6. Data are expressed as the average fold change±s.d., relative to non-irradiated cells (lysed in tandem) of three independent experiments. (**d**) A549 cells expressing the psi-miR-34 (WT or MT) reporters were transfected with siRNA and lysed 36 h after exposure to 4 Gy. Lysates were analysed for luciferase, pri-miR-34a, pre-miR-34a and mature miR-34a expression by RT–qPCR. Renilla was normalized to Firefly. Pri-miR-34a and pre-miR-34 were normalized to β-actin mRNA, mature miR-34a was normalized to U6. Graphed is the fold change±s.d., relative to non-irradiated cells; *n*=4 independent experiments. *P<0.05, one-tailed Student's *t*-test. (**e**) A549 cells transfected with 2′-*O*-methyl inhibitors were exposed to 6 Gy of IR. Cells were lysed at the indicated time post IR. Lysate was split and analysed for protein expression by western blot (top) and miR-34 expression (bottom) by RT–qPCR. Bands were quantificated using ImageJ.

We observed an existing and abundant pool of miR-34 present in four tested human cancer cell lines, before any DNA damage stimulus (Supplementary Fig. 1). To determine the role of this pool of existing miR-34, we generated a luciferase reporter system to measure miR-34 gene silencing activity (Supplementary Fig. 2). We defined activity as the level of suppression exerted on the reporter containing a fully complementary miR-34a target site (psi-miR-34, WT) compared with the level of suppression exerted on a control reporter containing a mutated miR-34 target site (psi-miR-34, MT). Transfection of the reporter system into cancer cell lines of different origins showed that the pool of existing miR-34 was inactive, as there was no suppression of the WT reporter compared with the MT (Fig. 1a). In contrast, our control reporter system (designed to measure *let-7a* activity, Supplementary Fig. 2) showed existing *let-7a* in cells was active (Fig. 1a and Supplementary Fig. 3). In contrast to the existing miR-34, exogenous miR-34-transfected into cells was able to suppress the WT reporter (Supplementary Fig. 4), suggesting that there was a difference between the existing pool of miR-34 and exogenous synthetic miR-34. Of note, transfection of exogenous miR-34a, miR-34b or miR-34c equally silenced the WT reporter, indicating that our system accurately measured all of the human miR-34 genes (Supplementary Fig. 5).

As our assay used to detect miR-34 could not determine whether the existing miR-34 was in a single-stranded- (mature) or double-stranded (precursor) state, we analysed the pool of miR-34 by native gel northern blot. We found that miR-34 migration was consistent with single-strand, mature miR-34 (Fig. 1b), which is the active form of other miRNAs, such as *let-7*. Furthermore, we were unable to detect the miR-34* strand by northern blot, as is found with other active miRNAs. These findings were consistent for miR-34a*/b*/c* across several cell lines (Supplementary Fig. 6) by reverse transcription–quantitative PCR(RT–qPCR). These findings confirmed that the pool of existing miR-34 in cells is in the mature, single-stranded state, yet, unlike other miRNAs in this form, appeared to be inactive.

### P53-independent activation of miR-34 following DNA damage

As miR-34 is critical in the DNA damage response^11^, we next investigated the impact of IR on the pool of inactive miR-34. To do this, we simultaneously measured miR-34 activity and expression before and after irradiation in A549 cells transfected with the psi-miR-34 reporters. Consistent with previous reports^4^,^5^,^6^, we found that miR-34 expression was induced following radiation exposure at 18 h (Fig. 1c, lines). Interestingly, however, we found that miR-34 activity began to increase as early as 6 h post irradiation (Fig. 1c, bars). These results suggested that IR potentially activated the pool of existing miR-34, before *de novo* miR-34 transcription and processing took place.

To confirm that the existing pool of miR-34 was activated by radiation, and to understand at what step activation was occurring, we inhibited *de novo* miR-34 expression at different steps in the process and measured miR-34 activity in each situation. We treated A549 cells containing the miR-34 reporters with small interfering RNA (siRNA) to *TP53*, *Drosha*, *Dicer*, *Argoanute2* or *GAPD*, exposed them to radiation, and then measured both miR-34 expression and activity. As expected, in cells without siRNA treatment, expression levels of pri-miR-34, pre-miR-34 and mature miR-34 RNAs were increased following exposure to IR, and this was consistent with an increase in miR-34 activity (Fig. 1d, black bars). However, although knockdown of p53 inhibited the increase in pri-, pre- and mature miR-34 expression, it only modestly decreased miR-34 activity (Fig. 1d, blue bars). Knockdown of Drosha attenuated pre- and mature miR-34 levels as expected, but again had only a modest effect on miR-34 activity (Fig. 1d, dark grey bars). Finally, although Dicer knockdown diminished mature miR-34 levels as expected, there still was only a modest decrease in miR-34 activity (Fig. 1d, red bars). In contrast, knockdown of Ago2 did not affect miR-34 expression, but there was almost complete inhibition of miR-34 activity (Fig. 1d, light grey bars). As a control, knockdown of glyceraldehyde-3-phosphate dehydrogenase (GAPD) had no effect on the miR-34 expression or activity (Fig. 1d, white bars). Similar results were found for miR-34b and miR-34c expression and activity (Supplementary Fig. 7), and western blot analysis confirmed protein knockdown (Supplementary Fig. 8). As an additional control, and also knowing that miR-17 levels do not change after radiation (Supplementary Fig. 9), we confirmed that knockdown of Drosha, Dicer and Ago2, but not p53, attenuated miR-17 activity in parallel with a reduction in expression (Supplementary Fig. 10). Based on these findings, it appeared that IR was activating the pool of existing miR-34, independent of *de novo* miR-34 transcription and/or processing, as inhibition of the creation of new miR-34 did not block IR-induced activity.

To confirm the functional activity of the radiation-activated existing miR-34 pool, we measured reduced expression of several previously confirmed miR-34 target genes, including CDK4 (refs 13, 14) and BCL2 (refs 15, 16). To do this, we irradiated cells pre-treated with anti-miR-34 or control 2′-*O*-methyl oligos and performed a time-course post-IR following gene expression. We found that both CDK4 and BCL2 expression was reduced quickly (within the first 12 h post IR) in the control-treated cells to approximately half of their initial levels (Fig. 1e). This repression continued through 48 h, with slightly enhanced repression (∼5–20%) starting at 24 h, at the time when *de novo* miR-34 expression increased (Fig. 1e, bottom bars). miR-34 inhibitor-treated cells confirmed that these target genes were primary regulated by miR-34. Our findings indicate that existing miR-34 is able to accomplish the majority of the desired gene expression reduction, quickly, within 6–12 h, before new miR-34 can be expressed/processed to reinforce suppression.

### DNA damage causes 5′-end phosphorylation of miR-34

**Figure 2:**
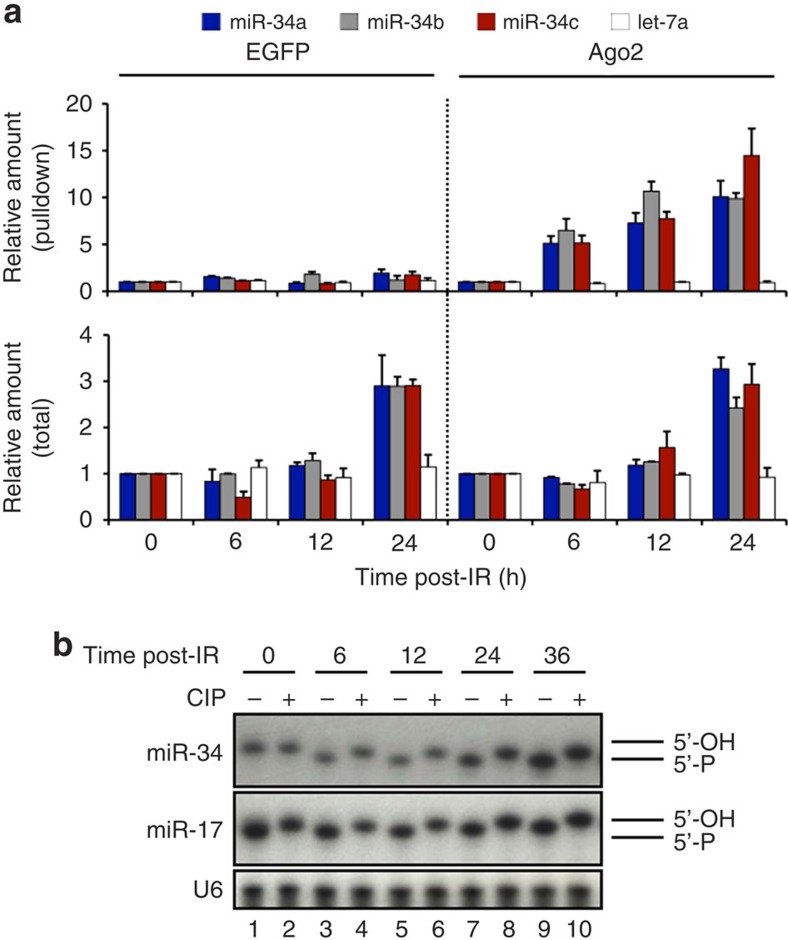
Quiescent mature miR-34 is not loaded into Ago2. (**a**) A549 cells expressing Flag/HA-tagged EGFP or Ago2 were exposed to 4 Gy and lysed at the indicated time. miRNA expression was analysed from Flag-immunoprecipitates (top) and total lysate (bottom) by RT–qPCR. Results were normalized to miR-17. Graphed is the fold change±s.d., relative to non-irradiated cells; *n*=4 independent experiments. (**b**) 50 μg of total RNA extracted from A549 cells exposed to 4 Gy was untreated or treated with CIP and separated by denaturing PAGE. RNA was detected by northern blot with ^32^P-labelled probes. U6 was used for normalization. Synthetic 5′OH and 5′P miR-34 from Dharmacon were used as size markers.

To begin to understand how the existing miR-34 was activated by radiation, we tested the hypothesis that the existing pool of miR-34 may not be loaded onto Ago2, and only loaded after IR. To test this, cells expressing Flag- and haemagglutinin-tagged (HA-tagged) Ago2 or EGFP (Supplementary Fig. 11) were exposed to 4 Gy of radiation, HA-tagged proteins were immunoprecipitated and miR-34 levels were analysed at different time points. We found that Ago2 immunoprecipitates showed a fivefold increase of Ago2-bound miR-34 at 6 h and a tenfold increase at 24 h post-IR (Fig. 2a, top), supporting that IR led to miR-34 loading into Ago2. As expected, analysis of RNA from the total (non-immunoprecipitated) fraction confirmed that new miR-34 expression increased only at the 24-h time point (Fig. 2a, bottom). Results were normalized to miR-17 (Supplementary Fig. 9). As expected, there was no association of miR-34 in EGFP immunoprecipitates, nor did radiation have any effect on the amount of Ago2-bound *let-7* (Fig. 2a white bars). These findings suggested that the existing pool of miR-34 was only loaded into Ago2 after IR, likely explaining the inactive state of the existing miR-34 pool.

We hypothesized that the reason for lack of Ago2 loading (and thus inactivity) of the existing miR-34 until after IR could be a lack of a 5′-phosphate, as it is believed that a 5′-phosphate is required for Ago2 loading of miRNAs^17^,^18^,^19^,^20^. Furthermore, crystallographic data show there are several amino acids in the Ago2 MID domain that directly contact the 5′-phosphate of a loaded miRNA^21^,^22^,^23^. Of note, this hypothesis would be consistent with our finding that exogenous, synthetic miR-34 is active in our assays, as it is as it is supplied with a 5′-phosphate. Therefore, to test our hypothesis, RNA from non-irradiated and irradiated cells was evaluated for the presence of a 5′-phosphate, by treating the RNA with calf intestinal phosphatase (CIP), which removes the 5′-phosphate, decreasing the apparent mobility of miR-34 by one nucleotide^17^ on a northern blot. Treatment of RNA extracted from non-irradiated cells with CIP did not alter the mobility of the existing miR-34, whereas CIP treatment of RNA extracted from irradiated cells showed decreased mobility of miR-34 by one nucleotide (Fig. 2b). These findings indicate the presence of a 5′-phosphate on miR-34 within just a few hours of IR. As a control, CIP treatment of miR-17 resulted in a mobility shift of miR-17 with or without radiation treatment of cells, as expected.

### ATM is required for miR-34 phosphorylation

**Figure 3:**
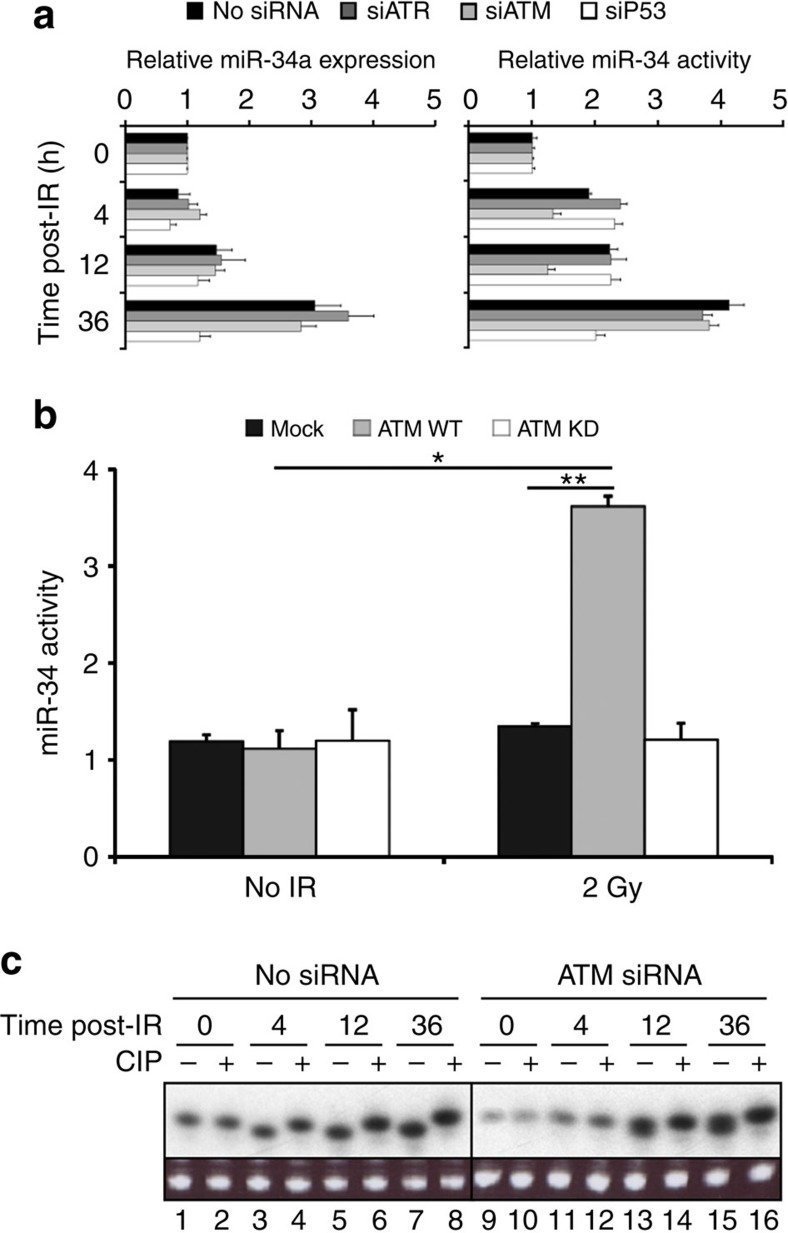
miR-34 5′-end phosphorylation is ATM-dependent following DNA damage. (**a**) A549 cells expressing psi-miR-34 (WT or MT) reporters were transfected with siRNA, exposed to 2 Gy and lysed at 12 h. Lysates were analysed for dual-luciferase activity. Renilla was normalized to Firefly and was expressed as the fold-suppression of MT/WT. Data are expressed as the fold change±s.d., relative to non-irradiated cells; *n*=2 independent experiments in triplicate. (**b**) ATM-deficient cells (GM16666) expressing wild-type ATM or a mutant (kinase dead) ATM were transfected with the psi-miR-34 (WT or MT) reporters. Cells were exposed to 2 Gy of IR and following a 4-h incubation, cells were analysed for miR-34 activity. Graphed is the average ±s.d. *n*=3 independent experiments. *P=0.035, two-tailed Student's *t*-test; **P=0.008, one-tailed Student's *t*-test. (**c**) 50 μg of total RNA extracted from A549 cells transfected with *ATM* siRNA or untransfected cells (from **a**) were untreated or treated with CIP and separated by denaturing PAGE. RNA was detected by northern blot with ^32^P-labelled probes. tRNA stained with ethidium bromide was used for normalization.

To understand how existing miR-34 could be phosphorylated post irradiation, we examined the role of two well-known kinases that are critical primary sensors in the DNA damage response, *ATM* and *ATR*^24^,^25^. To test their involvement, cells expressing the miR-34 reporters were treated with *ATM*, *ATR* or *TP53* siRNA. Cells were then treated with 2 Gy of radiation, lysed and analysed for both miR-34 activity and expression. Although *ATM* knockdown did not affect miR-34a expression (Fig. 3a, left panel), it strongly decreased early miR-34 activity (4 and 12 h), but not late miR-34 activity (which is due to new miR-34 transcription, 36 h; Fig. 3a, right panel). In contrast, *ATR* knockdown had no effect on miR-34a expression or activity at any time point. As expected, *TP53* knockdown reduced miR-34 expression, but cells retained early activity (4 and 12 h), which remained constant through the late (36 h) time point (Fig. 3a). We noted the same pattern with for miR-34b and miR-34c (Supplementary Fig. 12). These finding suggest that ATM is involved in the activation of existing miR-34 and its resulting early activity, but is not involved in the creation of new miR-34 at later time points.

To further confirm the role of ATM in existing miR-34 activation, we expressed wild-type ATM or a kinase-dead ATM mutant^26^ in ATM-deficient cells^27^ (Supplementary Fig. 13) transfected with the miR-34 reporters, and assayed them 4 h post IR (2 Gy). We found miR-34 early activity only in cells expressing wild-type ATM, and no miR-34 activity in cells transfected with the kinase-dead mutant, or in the ATM mutant parental cell line (Fig. 3b). To confirm that ATM was required specifically for the 5′-end phosphorylation of miR-34, RNA was extracted from non-irradiated and irradiated cells transfected with *ATM* siRNA and treated with CIP. Knockdown of ATM prevented 5′-end phosphorylation of miR-34 at the early (4 h) time point (Fig. 3c). At the later (12 and 36 h) time points, we saw the appearance of a doublet, which is likely consistent with *de novo* miR-34 transcription and processing, creating new 5′-phosphorylated miR-34. These findings indicate the requirement of ATM enzymatic activity for the 5′ phosphorylation of existing miR-34.

### hClp1 works with ATM to phosphorylate and activate miR-34

**Figure 4:**
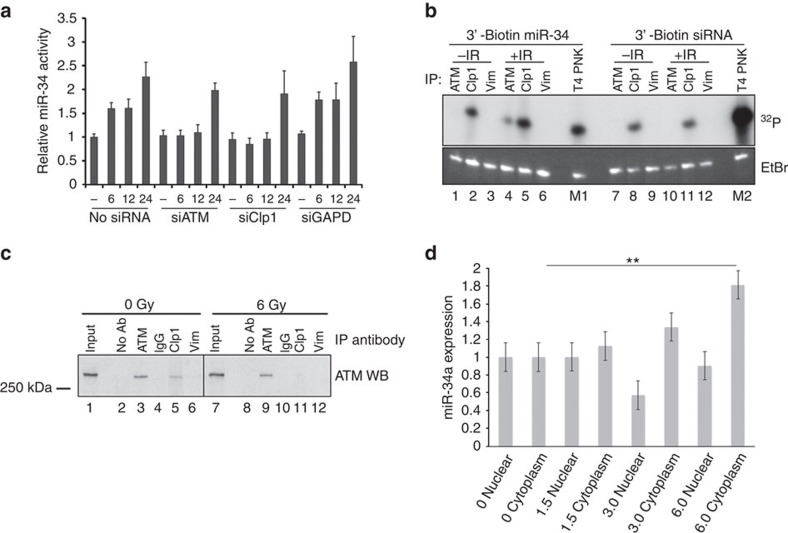
miR-34 activation, phosphorylation and localization. (**a**) A549 cells expressing psi-miR-34 (WT or MT) reporters were transfected with siRNA, exposed to 2 Gy and lysed at the indicated time. Lysates were analysed for luciferase activity and miR-34 expression by RT–qPCR. Renilla was normalized to Firefly and was expressed as the fold-suppression of MT/WT. MiR-34 expression was normalized to U6. Data are expressed as the fold change±s.d., relative to non-irradiated cells; *n*=4 independent experiments. (**b**) Untreated or irradiated (6 Gy) A549 cells were lysed and ATM, Clp1 or Vimentin were immunoprecipitated. Immunoprecipitates (or T4 PNK) were incubated with 3′-biotinylated RNAs and gamma-32P ATP (6,000 Ci mmol^−1^). RNA was selected using Avidin-D Agarose. RNA was eluted by phenol extraction, precipitated and analysed by 10% Urea-PAGE. Ethidium bromide-stained gel is shown as a loading control. (**c**) A549 cells were untreated or exposed to 6 Gy. Following a 3-h incubation, cells were lysed and 50 μg of lysate was incubated with 1 μg of ATM, IgG, Clp1 or Vimentin antibody (as indicated). Antibodies were captured with Protein A/G agarose and protein was eluted with SDS–PAGE sample buffer. Samples were separated on a 4–20% gradient gel and transferred to PVDF. ATM was probed for using anti-ATM antibody. The input control is 1/100th of the lysate used to IP. (**d**) Total RNA was isolated from nuclear and cytoplasmic fractions at different time points post irradiation with 4 Gy in A549 cells. miR-34 miRNA was measured and data are expressed as the average fold change±s.d. after normalization to miR-17. Samples were run in triplicate and the experiment was repeated four separate times. *P*-value is based on a two-tailed, two-sided *t*-test. **P=0.045.

To assess what other factors may be involved in the phosphorylation of miR-34, we investigated the role of the RNA kinase hClp1, which has previously been shown to rapidly phosphorylate transfected siRNAs bearing 5′-hydroxyls, as well as both double- and single-stranded siRNAs *in vitro*^28^. Although Clp1 has not been previously shown to phosphorylate miRNAs, it seemed logical that there could be a physiological role given its ability to phosphorylate siRNAs. We therefore knocked down hClp1 using siRNA (Supplementary Fig. 14), and evaluated miR-34 activity post irradiation. We found that hClp1 knockdown phenocopied ATM knockdown, resulting in loss of early miR-34 activation (Fig. 4a). We next tested if hClp1 was capable of phosphorylating the 5′end of miR-34 (and a control siRNA) in both non-irradiated and irradiated cells, by incubating immunoprecipitates of ATM, Clp1 or Vimentin with 3′-biotinylated RNAs and gamma-32P ATP (6,000 Ci mmol^−1^). We found that the hClp1 immunoprecipitate was capable of phosphorylating miR-34, and that this phosphorylation was enhanced by radiation, but was not radiation-dependent (Fig. 4b). Of note, hClp1 phosphorylation of the control siRNA was not altered by radiation. In contrast, we found that miR-34 phosphorylation by the ATM immunoprecipitate only took place with the addition of radiation. To further show the dependence of 5′-end phosphorylation of miR-34 on hClp1, RNA was extracted from non-irradiated and irradiated cells transfected with *hCLP1* siRNA and treated with CIP. Knockdown of hCLP1 prevented 5′-end phosphorylation of miR-34 (Supplementary Fig. 15), identical to what we found with siRNA to *ATM* (Supplementary Fig. 15 and Fig. 3c).

Because our findings suggested that hClp1 may be the kinase that leads to existing miR-34 phosphorylation and activation, we tested if hCLP1 interacted with or was a downstream target of ATM. We performed an immunoprecipitation of ATM and or hClp1^+/−^ radiation. We found that ATM and hClp1 definitively form a dynamic radiation-dependent complex; in the absence of radiation ATM and hClp1 interact with each other, however, after cells have been radiated, the interaction between ATM and Clp1 is significantly decreased (Fig. 4c and Supplementary Fig. 16). We next evaluated if ATM and Clp1 localize together in the cell. We found that both are primarily nuclear, both before and after radiation, and there is no apparent change in levels of either protein with irradiation (Supplementary Fig. 16).

Because it seemed that the proteins responsible for its phosphorylation resided in the nucleus, we measured nuclear and cytoplasmic miR-34 levels before and after radiation. We did find a significant increase in cytoplasmic miR-34 levels post irradiation, within 6 h after exposure to radiation (Fig. 4d). These findings support the possibility that ATM, Clp1 and unphosphorylated miR-34 could be localized together, in the nucleus, and that irradiation alters this complex, allowing this existing miR-34 to be released, exported and subsequently phosphorylated into its functional form.

As our findings represent a novel paradigm of immediate miRNA activation after radiation, we tested if other miRNAs might use a similar mechanism. We focused on miRNAs that appeared to fit a similar paradigm as miR-34, in that they are upregulated by radiation^29^, yet they are dispensable for development and viability in *C. elegans*, but these animal exhibit a hypersensitivity to radiation^30^,^31^. We therefore treated RNA from A549 cells with Terminator 5′ Phosphate-dependent exonuclease, which will digest phosphorylated miRNAs, and analysed the respective miRNAs by northern blot to look for digestion. Of the six miRNAs we assayed, we found that miR-34 (the positive control) was the only miRNA resistant to Terminator 5′ Phosphate-dependent exonuclease, whereas miR-17 (the negative control), miR-19, miR-24, miR-31 and miR-138 were susceptible to digestion, indicating they had a phosphorylated 5′end before radiation (Supplementary Fig. 17). Although this does not rule out the possibility that any other miRNAs use the same mechanism of early activation used by miR-34, it does suggest that this is not a widely used mechanism, and highlights the critical role of miR-34 in the rapid DNA damage response.

## Discussion

Here, we show for the first time a requirement for the 5′-end phosphorylation status of a miRNA, specifically miR-34, as being a critical determinant in its activity. Although the expression of many miRNAs are modulated by extracellular stimuli such as DNA damage^32^, inflammation^33^, receptor signalling^34^,^35^ and hypoxia^36^, this is the first evidence that a miRNA can exist in an inactive pool in cells, ready to mediate an early response mechanism. Although it is largely believed that miRNA biogenesis is coupled to Argonaute loading^37^,^38^, our work indicates that there may be exceptions for particular miRNAs, where coupling is dependent on an external stimulus leading to miRNA modification, such as phosphorylation. It appears possible that in the absence of genotoxic stress, miR-34 is transcribed, partly processed and sequestered somewhere in the cell, such as in the nucleus, until DNA damage, at which point it is shuttled^39^ into the cytoplasm and phosphorylated. This hypothesis is further supported by the ability of hClp1 to phosphorylate miR-34, without radiation, but the requirement of radiation for ATM, a DNA damage sensor, to enhance phosphorylatation of miR-34, and the interaction of ATM with hClp1 before radiation, and their separation upon radiation. Alternatively, miR-34 could be normally processed and then unphosphorylated, or miR-34 could be methylated, preventing initial phosphorylation^40^,^41^. Although further work is necessary to fully elucidate these mechanisms, what is clear is that miR-34 plays a unique and special role in managing the early DNA damage response.

There remain several unanswered questions, such as how cells differentiate between miR-34 and other miRNAs, and how ATM/hClp1 functions in this process. Further molecular, biochemical and functional approaches will help elucidate these mechanisms, opening numerous avenues for further understanding of miRNA regulation. Regardless, our work provides significant insight into a novel mechanism by which cells are prepared for a rapid response to DNA damage, with a pool of existing transcribed miR-34, waiting for rapid activation through phosphorylation. The activation mechanism discovered here can be thought of as analogous to posttranslational modifications required for protein activation, and is a clever cellular solution to a challenging situation, where using damaged DNA to create the tools for repair could be considered both inefficient and unwise.

## Methods

### Plasmids

The miR-34 WT, miR-34 MT, let-7 WT, let-7 MT, miR-17 WT, miR-17 MT dual luciferase reporter plasmids were generated by annealing and cloning synthetic oligos (Supplementary Table 1) into the *Xho*I and *Not*I restriction sites of psiCheck2 (Promega). Plasmids were sequenced following Maxi Prep (Qiagen). pIRESneo-FLAG-HA EGFP (10825), pIRESneo-FLAG-HA Ago2 (10822), pcDNA3.1(+)Flag-His-ATM wt (31985) and pcDNA3.1(+)Flag-His-ATM kd (31986) were purchased from Addgene.

### Cell lines and culture

A549 (CCL-185), Hela (CCL-2), MCF-7 (HBT-22) and H460 (HTB-177) cells were purchased from the American Type Culture Collection and cultured according to the manufacturer's protocol in media supplemented with 10% fetal bovine serum (Gibco), 100 U penicillin and 100 μg ml^−1^ streptomycin (Gibco). ATM-deficient (GM16666) cells were purchased from the Coriell Institute and cultured according to the manufacturer′s protocol in media supplemented with 2 mM L-glutamine, 15% fetal bovine serum and 100 μg ml^−1^ Hygromycin B.

### Irradiation

Radiations were performed using an XRAD 320 30 kVp, 12.5 mA (Precision X-Ray).

### Absolute quantification of miR-34 in cell lines

Cells in log growth phase on 10 cm plates were washed with PBS and lysed in the plate with 2.5 ml of TRIzol and total RNA was extracted. Concentration was assessed using a NanoDrop1000. MiR-34a, miR-34b and miR-34c were analysed from 1 and 10 ng of total RNA using MicroRNA TaqMan Assays (Applied Biosystems) according to the manufacturer's protocol. Standard curves for each miR-34 gene were generated by assaying synthetic RNA corresponding to each miR-34 gene with MicroRNA TaqMan Assays (Applied Biosystems). This experiment was performed twice, to generate biological replicate, with each sample being run in triplicate to generate technical replicates. All of the data were averaged together and the standard curves were generated by linear regression (*R*^2^ values for each slope are shown). Moles of each miR-34 gene were determined by back-calculating each Ct value to the corresponding curve for that gene.

### Analysis of miR-34 and let-7 activity in cancer cell lines

Hela, A549, H460 or MCF-7 cells were seeded in a 24-well plate at 50% confluency. Cells were transfected the following day with 10 ng of plasmid DNA using Lipofectamine 2000 (Invitrogen). Cells were washed with PBS and were lysed in wells with 100 μl of Passive Lysis Buffer (Promega) on ice for 30 min. Lysates were centrifuged at 16,000 *g* at 4 °C, for 15 min and the supernatant was transferred to a fresh tube. The Dual-luciferase Reporter Assay System (Promega) was used to analyse samples; read with a 2-s delay, 5 s read time in a Berthold Technologies Lumat LB 9507 single-read luminometer. Firefly and Renilla luciferase activities were measured by a two-point, quantitative titration of cell extract, the values of which are averaged together to generate one data point. Results are the average and standard deviation of two independent experiments performed in triplicate.

### Preparation of synthetic RNA duplexes

RNA duplexes were generated by annealing synthetic RNAs (synthesized with 5′-phosphates, Dharmacon) in a reaction containing Buffer A (50 mM Tris, pH 8.0, 250 mM NaOAc, 2 mM MgCl2). After heating at 95 °C for 10 min, the reactions were cooled to 4 °C. RNA was precipitated and separated on a 10% native gel. Duplexes were gel purified and reconstituted at a concentration of 100 μM in Buffer A, determined by gel quantification. RNA duplexes were aliquoted and stored at −80 °C for use.

### Co-transfection of cell lines with synthetic RNA duplexes

A549 cells were seeded in a 24-well plate at 50% confluency. Cells were transfected the following day with 10 ng of plasmid DNA and synthetic RNA duplexs (as indicated) using Lipofectamine 2000 (Invitrogen). Following a 16-h incubation, the cells were washed with PBS and were lysed in wells with 100 μl of Passive Lysis Buffer (Promega) on ice for 30 min. Lysates were centrifuged at 16,000 x*g* at 4 °C, for 15 min and the supernatant was transferred to a fresh tube. The Dual-luciferase Reporter Assay System (Promega) was used to analyse samples; read with a 2-s delay, 5 s read time in a Berthold Technologies Lumat LB 9507 single-read luminometer. Firefly and Renilla luciferase activities were measured by a two-point, quantitative titration of cell extract, the values of which are averaged together to generate one data point. Results are the average and standard deviation of two independent experiments performed in duplicate.

### MiR-34 and miR-34* northern blot

A549 cells plated at 50% confluency in 10 cm plates were exposed to 6 Gy. At the indicated time, cells were washed with PBS and lysed in wells with TRIzol. Total RNA was extracted as per Rio *et al.*^42^ and 50 μg pellets were resuspended in native gel loading buffer. Samples were separated on a Criterion 15% TBE Precast Gel (Bio-Rad) run in 1 × TBE. The gel was stained with ethidium bromide and RNA was transferred to Hybond-N+ nylon (Amersham). RNA was crosslinked using a ultraviolet Stratalinker 2400 on the optimal crosslink setting. MiR-34a and miR-34a* were probed using complimentary 5′-end-labelled DNA probes according to the Bartel Lab (original) northern blot protocol. 10 fmol of synthetic single- and double-stranded miR-34 were run as size markers.

### Analysis of miR-34/miR-34* in cell lines

HeLa, A549, H460 and MCF-7 cells in log growth phase were washed with PBS and lysed in wells with TRIzol according to the study by Rio *et al.*^1^ 25 ng of total RNA was analysed using MicroRNA TaqMan Assays (Applied Biosystems) directed against each gene as indicated. Results were normalized to U6 RNA. Ratios were calculated using the delta-Ct values for each miRNA/miRNA*, respectively. Expression of miR-34b* was below the limit of quantification in MCF-7 cells (40-cylces) and thus that samples was labelled ‘Undetermined'.

### Analysis of miR-34 expression and activity after IR

A549 cells were seeded in a 60-mm plate at 80% confluency. The following day, cells were transfected with 1 μg of plasmid DNA using Lipofectamine 2000. After transfection, the cells were re-plated in a 24-well plate at 50% confluency. After 12 h the cells were exposed to 6 Gy. At the indicated time, cells were washed with PBS and lysed in wells using Passive Lysis Buffer (Promega) for 30 min on ice. Lysates were split; half used for luciferase analysis (as described above), half used for RNA analysis. To do so, total RNA was extracted as per the study by Rio *et al.*^43^ and 25 ng of total RNA was analysed using the (Applied Biosystems) microRNA TaqMan Assays according to the manufacture's protocol. Results were normalized to U6 RNA and miR-34 expression, relative to non-irradiated cells was calculated using the delta-delta Ct method. A duplicate plate, not exposed to IR was used as a baseline for each time point. Results are the average and standard deviation of two independent experiments performed in triplicate.

### Analysis of miR-34a/b/c in siRNA-treated cells after IR

A549 cells were seeded in 10 cm plates at 80% confluency. The following day, cells were transfected with 1 μg of plasmid DNA (psi-miR-34 WT or psi-miR-34 MT) and 200 pmol ON-TARGETplus siRNA SmartPool (Dharmacon) using Lipofectamine 2000 as indicated. After transfection, the cells were re-plated in a six-well plate at 50% confluency. After a 12-h incubation, the cells were exposed to 6 Gy of IR. Following a 36-h incubation, cells were washed with PBS and lysed in wells with Passive Lysis Buffer (Promega). 25 μl of lysate was withdrawn for luciferase analysis (described in protocol above); total RNA was extracted from 100 μl of lysate using TRIzol; 75 μl of lysate was boiled in SDS Sample buffer and analysed using western blot analysis (shown in Supplementary Fig. 8, described below). Primary miR-34b/c, mature miR-34b and mature miR-34c was analysed using MicroRNA TaqMan Assays (Applied Biosystems) according to the manufacturer's protocol. Pri- miR-34b/c was normalized to beta-Actin mRNA; mature miR-34b and mature miR-34c were normalized to U6 RNA. Ct-values were analysed using the delta-delta Ct method. Results are the average and standard deviation of two independent experiments.

### Analysis of protein knockdown by western blot

Lysates from cells transfected with luciferase reporter plasmid DNA and siRNA were boiled in Laemmli Sample Buffer (Bio-Rad). Samples were separated on a 4–20% Criterion TGX Precast Gel (Bio-Rad). Protein was transferred to Whatman BA85 Nitrocellulose and protein was detected using the following antibodies: anti-Vimentin V9 (ab8069, Abcam, 1:10,000), anti-GAPDH 6C5 (ab8245, Abcam, 1:50,000), anti-Drosha (ab12286, Abcam, 1:1,000), anti-Dicer 13D6 (ab14601, Abcam, 1:1,000), Anti-Ago2 11A9 (SAB4200085, Sigma, 1:1,000). Primary antibodies were detected using the following horseradish peroxidase (HRP)-conjugated secondary antibodies: sheep anti-mouse IgG, HRP-linked Ab (NA931, GE Healthcare, 1:50,000), goat anti-rat IgG, HRP-linked Ab (NA935, GE Healthcare, 1:50,000), donkey anti-rabbit IgG, HRP-linked Ab (NA934, GE Healthcare, 1:50:000). Pierce ECL western blotting substrate (PI-32109) was used for detection.

### Analysis of miR-17 in siRNA-treated irradiated cells

A549 cells were seeded in a 60-mm plate at 80% confluency. The following day, cells were transfected with 1 μg of plasmid DNA (psi-miR-17 WT or psi-miR-17 MT) and 200 pmol ON-TARGETplus siRNA SmartPool (Dharmacon) using Lipofectamine 2000 as indicated. After transfection, the cells were re-plated in a six-well plate at 50% confluency. After a 12-h incubation, the cells were exposed to 6 Gy of IR. Following a 36-h incubation, cells were washed with PBS and lysed in wells with Passive Lysis Buffer (Promega). 25 μl of lysate was withdrawn for luciferase analysis (described in protocol above); total RNA was extracted from 100 μl of lysate using TRIzol as per the study by Rio *et al.*^1^ Primary miR-17, pre-miR-17 and mature miR-17 RNA were analysed using MicroRNA TaqMan Assays (Applied Biosystems) according to the manufacturer's protocol. Pri-miR-17 and pre-miR-17 were normalized to β-Actin mRNA; mature miR-17 was normalized to U6 RNA. Ct-values were analysed using the delta-delta Ct method. Results are the average and standard deviation of two independent experiments.

### miR-34 target mRNA expression analysis following IR

A549 cells were seeded in 10 cm plates at 80% confluency. The following day cells were transfected with 500 pmol 2′-*O*-methyl inhibitors (Ambion) directed against either miR-34 or miR-20 (labelled as Control Inhibitor). After transfection, the cells were re-plated in a six-well plate at 50% confluency. The following day cells were exposed to 6 Gy of IR and were lysed in wells at the indicated time using Passive Lysis Buffer (Promega). Cleared lysates were divided into two parts for RNA analysis (described above) and western blot analysis. CDK4, BCL2 and α-tubulin were probed using the following primary antibodies: CDK4 (Cell Signaling, #12790, 1:1,000); Bcl-2 (Cell Signaling, #2872, 1:1,000); α-tubulin (Abcam, ab15246, 1:25,000).

### Analysis of FLAG-HA-tagged EGFP and AGO2 expression in cells

A549 cells in log growth phase were plated on a 10-cm plate at 80% confluency and calcium-phosphate transfected with 5 μg of plasmid DNA per plate. Following a 24-h incubation, the cells were exposed to 4 Gy of ionizing radiation. Cells were harvested at 6, 12 and 24 h post IR. For the 0-h time point, cells were harvested in tandem with the 24-h time point. Plates were washed with PBS and the cells were lysed in wells with Buffer A (10 mM Tris, pH 8.0, 1 mM EDTA pH 8.0, 0.1 mM MgCl2, 100 mM NaCl, 1% Triton X-100) at 4 °C for 20 min. Lysates were cleared by centrifugation at 16,000*g* for 20 min at 4 °C. One-fifth of the lysate was withdrawn for western blot analysis. Lysate was boiled in SDS Sample buffer and samples were separated using a 4–20% Criterion TGX Precast Gel (Bio-Rad). Protein was transferred to Whatman BA85 Nitrocellulose and protein was detected using: mouse anti-FLAG mAb (F3165, Sigma, 1:10,000) and sheep anti-mouse IgG, HRP-linked Ab (NA931, GE Healthcare, 1:50,000). Pierce ECL western blotting substrate (PI-32109) was used for detection.

### Co-immunoprecipitation of miR-34 and AGO2

A549 cells grown in 10 cm plate were calcium-phosphate transfected with 5 μg of plasmid DNA per plate. Following a 24-h incubation, the cells were exposed to 4 Gy. Cells were harvested at 6, 12 and 24 h post IR. For the 0 h time point, cells were harvested in tandem with the 24 h time point. Plates were washed with PBS and the cells were lysed in wells with Buffer A (10 mM Tris, pH 8.0, 1 mM EDTA, pH 8.0, 0.1 mM MgCl_2_, 100 mM NaCl, 1% Triton X-100) at 4 °C for 20 min. Lysates were cleared by centrifugation at 16,000*g* for 20 min at 4 °C. One-fifth of the lysate was withdrawn for western blot analysis (Supplementary Fig. 11) and one-fifth of the cell lysate was withdrawn for total RNA analysis (described above). Anti-Flag M2 agarose beads (A2220, Sigma) were washed with 10 × volume of PBS twice, then once with Buffer A and incubated with lysate for 3 h at 4 °C. The samples were washed twice with Buffer A, twice with Buffer A containing 400 mM NaCl and once with PBS. Beads were incubated with proteinase K and then extracted with phenol–chloroform to isolate co-precipitated RNA. RNA was analysed as described above.

### 5′-Phosphase analysis of miR-34 in siRNA-treated cells

A549 cells in log growth phase were plated at 80% confluency in 10 cm plates. Cells were transfected with 1 nmol ON-TARGETplus siRNA SmartPool (Dharmacon) using Lipofectamine 2000 as indicated. Following a 24-h incubation, the cells were exposed to 2 Gy of IR. At 4 and 12 h post-IR cells were washed with PBS and lysed in wells with 2.5 ml of TRIzol; no-IR (0-time point) was harvested in tandem with the 12 h time point. Total RNA was extracted as per the study by Rio *et al.*^1^ 50 μg of RNA was incubated with 10 U of CIP (New England Biolabs) at room temperature for 15 min. Samples were extracted with phenol, ethanol precipitated and resuspended in formamide loading buffer. Samples were separated on a 15% TBE-Urea Criterion Precast Gel (Bio-Rad). The gel was stained with ethidum bromide (used as loading control) and the RNA was transferred to Nylon Hybond-N+ (Amersham). RNA was crosslinked in a Stratagene UV Stratalinker 2400 run on the ‘optimal crosslink' setting. miR-34 northern blot was performed as previously described in the Bartel Lab (original) Northern Blotting Protocol using a DNA probe complementary to miR-34a.

### Analysis of ATM on miR-34 activity

ATM-deficient GM16666 cells in log growth phase were plated on a 10-cm plate at 80% confluency and calcium-phosphate co-transfected with 5 μg of pcDNA3.1(+)Flag-His-ATM wt or pcDNA3.1(+)Flag-His-ATM kd plasmid DNA per plate and 100 ng of psi-miR-34 WT or psi-miR-34 MT^43^. Following a 24-h incubation, the cells were exposed to 2 Gy of ionizing radiation. Cells were harvested at 6 h post IR. Plates were washed with PBS and the cells were lysed in wells with Passive Lysis Buffer for 30 min on ice. Lysates were centrifuged at 16,000 *g* at 4 °C, for 15 min and the supernatant was transferred to a fresh tube. 20 μl of lysate was reserved for luciferase reporter activity (as described above); 80 μl of lysate was boiled in SDS Sample buffer and samples were separated using a 4–20% Criterion TGX Precast Gel (Bio-Rad). Protein was transferred to Whatman BA85 Nitrocellulose and protein was detected using: mouse anti-FLAG mAb (F3165, Sigma, 1:10,000) and sheep anti-mouse IgG, HRP-linked Ab (NA931, GE Healthcare, 1:50,000). Pierce ECL western blotting substrate (PI-32109) was used for detection (shown in Supplementary Fig. 11).

### ATM and Clp1 knockdown on miR-34 activity following IR

A549 cells were seeded in a 60-mm plate at 80% confluency. The following day, cells were transfected with 1 μg of plasmid DNA (psi-miR-34 WT or psi-miR-34 MT) and 200 pmol ON-TARGETplus siRNA SmartPool (Dharmacon) using Lipofectamine 2000 as indicated. After transfection, the cells were re-plated in a six-well plate at 50% confluency. After a 12-h incubation, the cells were exposed to 2 Gy of IR. At 4, 12 and 24 h post IR, cells were washed with PBS and lysed in wells with Passive Lysis Buffer (Promega). 25 μl of lysate was withdrawn for luciferase analysis (described above); 75 μl of lysate was boiled in SDS Sample buffer and samples were separated using a 4–20% Criterion TGX Precast Gel (Bio-Rad). Protein was transferred to Whatman BA85 Nitrocellulose and protein was detected using: anti-ATM 2C1 (1A1) (ab78, Abcam, 1:1,000), anti-Clp1 N3C3 (GTX115518, GeneTex, 1:1,000) and sheep anti-mouse IgG, HRP-linked Ab (NA931, GE Healthcare, 1:50,000) and HRP-conjugated anti-rabbit (ab6721, Abcam, 1:25,000). Pierce ECL western blotting substrate (PI-32109) was used for detection.

### *In vitro* kinase assay

A549 cells either untreated or exposed to 6 Gy were resuspended in 5 × volume of hypotonic buffer (50 mM Tris, pH 7.5, 10 mM KCl, 5 mM dithiothreitol). Swollen cells were homogenized and KCl, MgCl_2_ and glycerol were added to final concentrations of 100 mM, 5 mM and 10%, respectively. The homogenate was centrifuged at 500 *g* for 15 min. The supernatant was removed and the pellet was resuspended in Buffer B (50 mM Tris, pH 7.5, 2 mM MgCl_2_, 5 mM dithiothreitol, 10% glycerol). KCl was added to a final concentration of 400 mM and the homogenate was centrifuged at 10,000*g* for 15 min. The resultant nuclear extract was pre-cleared with Protein A/G Agarose equilibrated in Buffer B. 1 μg of ATM 2C1 (GeneTex), Clp1 8D5 (Novus), Vimentin V9 (Abcam) were incubated in 1 mg of extract. Protein A/G Agarose was added and the resultant immunoprecipitates were washed with Buffer B. 100 pmol of 3′-biotinylated miR-34 or 3′-biotinylated control siRNA were incubated with 25 μl of immunoprecipate beads in Buffer B and 0.1 μCi of 6,000 Ci mmol^−1^ gamma-32P-ATP was added. Reactions were incubated for 15 min at room temperature and 25 μl of Avidin-D Agarose (Vector Labs) was added. Avidin-D Agarose was washed with Buffer B+500 mM NaCl and RNA was extracted with phenol. RNA pellets were resuspended in formamide loading buffer and separated on a Criterion 10% TBE-Urea Precast Gel (Bio-Rad). The gel was stained with ethidium bromide and then fixed in 10% acetic acid, backed with DE81 paper and dried. The gel was exposed to Kodak BioMax MS film for 2 h.

### Immunoprecipitation with CLP1 and probing for ATM

A549 cells were treated with 4 Gy IR and whole-cell lysates were isolated at 0 (no IR), 45 min and 3 h after IR and crosslink IP were performed using Pierce Crosslink Immunoprecipitation kit (Pierce) using the manufacture's protocol. Briefly, 10 μg of ATM 2C1 (GTX70103, GeneTex) or CLP1 N3C3 (GTX115518 GeneTex) antibody were incubated with protein AG plus agarose for 60 min at room temperature followed by crosslinking the bound antibody to Protein A/G plus agarose with DSS crosslinker. 1 mg of pre-cleared lysate was added to the antibody-crosslinked resin and incubated overnight at 4 °C. The resin was washed to remove unbound proteins and then immunoprecipitated proteins were eluted using elution buffer. Eluted proteins were analysed by western blotting using anti-ATM Y170 (Genetex GTX61188 at 1:1,000) and anti-CLP1 N3C3 (GeneTex GTX115518 at 1:1,000).

### Analysis of nuclear and cytoplasmic fractionated proteins

A549 cells in log growth phase were irradiated with 2 Gy. Nuclear and cytoplasmic protein fractions were separated using the NE- PER Nuclear and Cytoplasmic Extraction Kit (Thermo Scientific) at 30 min and 3 h post IR; the 0-h time point (no-IR) was lysed in tandem with the 3 h time point. 50 μg of total protein was boiled in SDS Sample buffer and separated on a 4–20% TGX Criterion Precast Gel (Bio-Rad). Protein was transferred to polyvinylidene difluoride membrane (Millipore) and proteins were probed using the following antibodies: anti-ATM 2C1 (1A1) (ab78, Abcam, 1:1,000), anti-Clp1 N3C3 (GTX115518, GeneTex, 1:1,000), anti-p48 5E10 (ab487, Abcam, 1:1,000), anti-GAPDH 6C5 (ab8245, Abcam, 1:20,000) and sheep anti-mouse IgG, HRP-linked Ab (NA931, GE Healthcare, 1:50,000) and HRP-conjugated anti-rabbit (ab6721, Abcam, 1:25,000). Pierce ECL western blotting substrate (PI-32109) was used for detection.

### Cytoplasmic and nuclear miRNA RT–qPCR

A549 cells were irradiated with 4 Gy and total RNA was isolated from the nuclear and cytoplasmic fractions separately using Cytoplasmic and Nuclear RNA purification kit (Norgen Biotek Corp.) at 0 (no IR), 1.5 h and 3.0 h post IR. Target RNAs were analysed by RT–qPCR using TaqMan microRNA assays (Applied Biosystems) according to the manufacture's protocol. Data were analysed using the delta-delta Ct method.

### 5′-End analysis of microRNAs

A549 cells in log growth phase were washed with PBS and lysed in wells with TRIzol. Total RNA was extracted as per the study by Rio *et al.*^1^ 50 μg of total RNA was treated with 10 U of Terminator 5′-Phosphate Dependent Exonuclease (Epicentre). Samples were extracted with phenol, ethanol precipitated and resuspended in formamide loading buffer. Samples were separated on a 15% TBE-Urea Criterion Precast Gel (Bio-Rad). The gel was stained with ethidum bromide (used as loading control) and the RNA was transferred to Nylon Hybond-N+ (Amersham). RNA was crosslinked in a Stratagene UV Stratalinker 2400 run on the ‘optimal crosslink' setting. Northern blot was performed as previously described in the Bartel Lab (original) Northern Blotting Protocol using a DNA probes complementary to the indicated miRNAs.

### Uncropped blots

Uncropped images of all blots in the main manuscript are shown in Supplementary Fig. 18.

## Additional information

**How to cite this article**: Salzman, D. W. *et al.* miR-34 activity is modulated through 5′-end phosphorylation in response to DNA damage. *Nat. Commun.* 7:10954 doi: 10.1038/ncomms10954 (2016).

## Supplementary Material

Supplementary Information Supplementary Figures 1-18 and Supplementary Tables 1-2

## References

[b1] Newman M. A. & Hammond S. M. Emerging paradigms of regulated microRNA processing. Genes Dev. 24, 1086–1092 (2010). 10.1101/gad.1919710 PMC2878647 20516194

[b2] Krol J., Loedige I. & Filipowicz W. The widespread regulation of microRNA biogenesis, function and decay. Nat. Rev. Genet. 11, 597–610 (2010). 20661255 10.1038/nrg2843

[b3] Kelnar K., Peltier H., Leatherbury N., Stoudemire J. & Bader A. G. Quantification of therapeutic miRNA mimics in whole blood from nonhuman primates. Anal. Chem. 86, 1534–1542 (2014). 24397447 10.1021/ac403044t PMC3982984

[b4] Chang T. C. *et al.* Transactivation of miR-34a by p53 broadly influences gene expression and promotes apoptosis. Mol. Cell 26, 745–752 (2007). 17540599 10.1016/j.molcel.2007.05.010 PMC1939978

[b5] He L. *et al.* A microRNA component of the p53 tumour suppressor network. Nature 447, 1130–1134 (2007). 17554337 10.1038/nature05939 PMC4590999

[b6] Raver-Shapira N. *et al.* Transcriptional activation of miR-34a contributes to p53-mediated apoptosis. Mol. Cell 26, 731–743 (2007). 17540598 10.1016/j.molcel.2007.05.017

[b7] Hermeking H. The miR-34 family in cancer and apoptosis. Cell Death Differ. 17, 193–199 (2009). 19461653 10.1038/cdd.2009.56

[b8] Miska E. A. *et al.* Most *Caenorhabditis elegans* microRNAs are individually not essential for development or viability. PLoS Genet. 3, e215 (2007). 18085825 10.1371/journal.pgen.0030215 PMC2134938

[b9] Park C. Y. *et al.* A resource for the conditional ablation of microRNAs in the mouse. Cell Rep. 1, 385–391 (2012). 22570807 10.1016/j.celrep.2012.02.008 PMC3345170

[b10] Leung A. K. & Sharp P. A. MicroRNA functions in stress responses. Mol. Cell 40, 205–215 (2010). 20965416 10.1016/j.molcel.2010.09.027 PMC2996264

[b11] Kato M. *et al.* The mir-34 microRNA is required for the DNA damage response *in vivo* in *C. elegans* and *in vitro* in human breast cancer cells. Oncogene 28, 2419–2424 (2009). 19421141 10.1038/onc.2009.106 PMC2941141

[b12] Burke S., Hammell M. & Ambros V. Robust distal tip cell pathfinding in the face of temperature stress is ensured by two conserved microRNAs in *Caenorhabditis elegans*. Genetics 200, 1201–1218 (2015). 26078280 10.1534/genetics.115.179184 PMC4574240

[b13] Bandi N. & Vassella E. miR-34a and miR-15a/16 are co-regulated in non-small cell lung cancer and control cell cycle progression in a synergistic and Rb-dependent manner. Mol. Cancer 10, 55 (2011). 21575235 10.1186/1476-4598-10-55 PMC3120797

[b14] Lal A. *et al.* Capture of microRNA-bound mRNAs identifies the tumor suppressor miR-34a as a regulator of growth factor signaling. PLoS Genet. 7, e1002363 (2011). 22102825 10.1371/journal.pgen.1002363 PMC3213160

[b15] Ji Q. *et al.* Restoration of tumor suppressor miR-34 inhibits human p53-mutant gastric cancer tumorspheres. BMC Cancer 8, 266 (2008). 18803879 10.1186/1471-2407-8-266 PMC2564978

[b16] Bommer G. T. *et al.* p53-mediated activation of miRNA34 candidate tumor-suppressor genes. Curr. Biol. 17, 1298–1307 (2007). 17656095 10.1016/j.cub.2007.06.068

[b17] Hutvagner G. *et al.* A cellular function for the RNA-interference enzyme Dicer in the maturation of the let-7 small temporal RNA. Science 293, 834–838 (2001). 11452083 10.1126/science.1062961

[b18] Basyuk E., Suavet F., Doglio A., Bordonne R. & Bertrand E. Human let-7 stem-loop precursors harbor features of RNase III cleavage products. Nucleic Acids Res. 31, 6593–6597 (2003). 14602919 10.1093/nar/gkg855 PMC275551

[b19] Zhang H., Kolb F. A., Jaskiewicz L., Westhof E. & Filipowicz W. Single processing center models for human Dicer and bacterial RNase III. Cell 118, 57–68 (2004). 15242644 10.1016/j.cell.2004.06.017

[b20] Rivas F. V. *et al.* Purified Argonaute2 and an siRNA form recombinant human RISC. Nat. Struct. Mol. Biol. 12, 340–349 (2005). 15800637 10.1038/nsmb918

[b21] Ma J. B. *et al.* Structural basis for 5'-end-specific recognition of guide RNA by the A. fulgidus Piwi protein. Nature 434, 666–670 (2005). 15800629 10.1038/nature03514 PMC4694588

[b22] Schirle N. T. & MacRae I. J. The crystal structure of human Argonaute2. Science 336, 1037–1040 (2012). 22539551 10.1126/science.1221551 PMC3521581

[b23] Elkayam E. *et al.* The structure of human argonaute-2 in complex with miR-20a. Cell 150, 100–110 (2012). 22682761 10.1016/j.cell.2012.05.017 PMC3464090

[b24] Shiloh Y. ATM and ATR: networking cellular responses to DNA damage. Curr. Opin. Genet. Dev. 11, 71–77 (2001). 11163154 10.1016/s0959-437x(00)00159-3

[b25] Jackson S. P. & Bartek J. The DNA-damage response in human biology and disease. Nature 461, 1071–1078 (2009). 19847258 10.1038/nature08467 PMC2906700

[b26] Canman C. E. *et al.* Activation of the ATM kinase by ionizing radiation and phosphorylation of p53. Science 281, 1677–1679 (1998). 9733515 10.1126/science.281.5383.1677

[b27] Ziv Y. *et al.* Recombinant ATM protein complements the cellular A-T phenotype. Oncogene 15, 159–167 (1997). 9244351 10.1038/sj.onc.1201319

[b28] Weitzer S. & Martinez J. The human RNA kinase hClp1 is active on 3' transfer RNA exons and short interfering RNAs. Nature 447, 222–226 (2007). 17495927 10.1038/nature05777

[b29] Weidhaas J. B. *et al.* MicroRNAs as potential agents to alter resistance to cytotoxic anticancer therapy. Cancer Res. 67, 11111–11116 (2007). 18056433 10.1158/0008-5472.CAN-07-2858 PMC6070379

[b30] Weidhaas J. B., Eisenmann D. M., Holub J. M. & Nallur S. V. A *Caenorhabditis elegans* tissue model of radiation-induced reproductive cell death. Proc. Natl Acad. Sci. USA 103, 9946–9951 (2006). 16788064 10.1073/pnas.0603791103 PMC1502559

[b31] Weidhaas J. B., Eisenmann D. M., Holub J. M. & Nallur S. V. A conserved RAS/mitogen-activated protein kinase pathway regulates DNA damage-induced cell death postirradiation in Radelegans. Cancer Res. 66, 10434–10438 (2006). 17079464 10.1158/0008-5472.CAN-06-2182

[b32] Zhang X., Wan G., Berger F. G., He X. & Lu X. The ATM kinase induces microRNA biogenesis in the DNA damage response. Mol. Cell 41, 371–383 (2012). 21329876 10.1016/j.molcel.2011.01.020 PMC3114434

[b33] O'Connell R. M., Rao D. S. & Baltimore D. microRNA regulation of inflammatory responses. Annu. Rev. Immunol. 30, 295–312 (2012). 22224773 10.1146/annurev-immunol-020711-075013

[b34] Nishida N., Mimori K., Mori M. & Calin G. A. EGFR gets in the way of microRNA biogenesis. Cell Res. 23, 1157–1158 (2013). 23835474 10.1038/cr.2013.87 PMC3790241

[b35] Shen J. *et al.* EGFR modulates microRNA maturation in response to hypoxia through phosphorylation of AGO2. Nature 497, 383–387 (2013). 23636329 10.1038/nature12080 PMC3717558

[b36] Crosby M. E., Kulshreshtha R., Ivan M. & Glazer P. M. MicroRNA regulation of DNA repair gene expression in hypoxic stress. Cancer Res. 69, 1221–1229 (2009). 19141645 10.1158/0008-5472.CAN-08-2516 PMC2997438

[b37] Kim Y. & Kim V. N. MicroRNA factory: RISC assembly from precursor microRNAs. Mol. Cell 46, 384–386 (2012). 22633486 10.1016/j.molcel.2012.05.012

[b38] Kawamata T. & Tomari Y. Making RISC. Trends Biochem. Sci. 35, 368–376 (2010). 20395147 10.1016/j.tibs.2010.03.009

[b39] Wan G. *et al.* DNA Damage-induced nuclear export of precursor microRNAs is regulated by the ATM-AKT pathway. Cell Rep. 3, 2100–2112 (2013). 23791529 10.1016/j.celrep.2013.05.038 PMC3796289

[b40] Xhemalce B., Robson S. & Kouzarides T. Human RNA methyltransferase BCDIN3D regulates microRNA processing. Cell 151, 278–288 (2012). 23063121 10.1016/j.cell.2012.08.041 PMC3640255

[b41] Xie M. *et al.* Mammalian 5'-capped microRNA precursors that generate a single microRNA. Cell 155, 1568–1580 (2013). 24360278 10.1016/j.cell.2013.11.027 PMC3899828

[b42] Rio D. C., Ares M. Jr., Hannon G. J. & Nilsen T. W. Purification of RNA using TRIzol (TRI reagent). Cold Spring Harb. Protoc. 2010, pdb prot5439 (2010). 20516177 10.1101/pdb.prot5439

[b43] Meister G. *et al.* Human Argonaute2 mediates RNA cleavage targeted by miRNAs and siRNAs. Mol. Cell 15, 185–197 (2004). 15260970 10.1016/j.molcel.2004.07.007

