# Supplementary material for: miR-34 activity is modulated through 5′-end phosphorylation in response to DNA damage
Source: Nat Commun. 2016 Mar 21;7:10954. doi: 10.1038/ncomms10954 (PMC4802117; doi:10.1038/ncomms10954)
Supplement: Supplementary Information — Supplementary Figures 1-18 and Supplementary Tables 1-2 [file ncomms10954-s1.pdf]

Supplementary Fig. 1

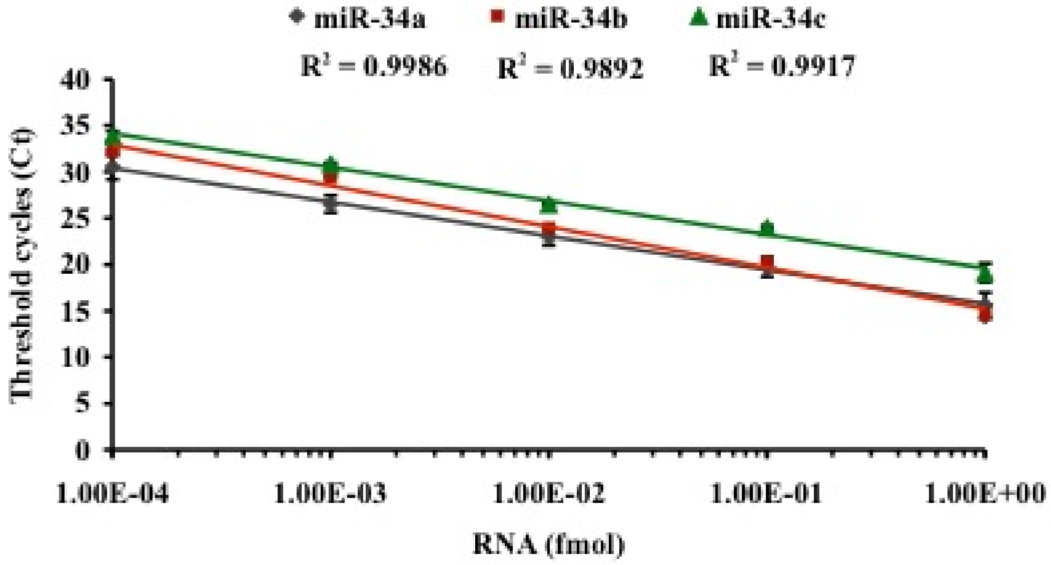

Cell line	Isoform	C_t/1ng$_{total\ RNA}$	C_t/10ng$_{total\ RNA}$	Amount/ng$_{total\ RNA}$
HeLa	miR-34a	20.1	17.2	25amol
	miR-34b	30.5	28.3	5amol
	miR-34c	31.1	28.8	2amol
A549	miR-34a	23.1	19.2	10amol
	miR-34b	25.6	23.2	25amol
	miR-34c	30.6	28.5	2amol
H460	miR-34a	21.4	19.7	15amol
	miR-34b	30.7	27.3	5amol
	miR-34c	31.8	29.2	1amol
MCF-7	miR-34a	22.6	19.7	15amol
	miR-34b	29.3	26.5	5amol
	miR-34c	29.8	26.6	5amol

Supplementary Figure 1: Quantification of miR-34 in cancer cell lines. Standard curves were generated by RT-qPCR on synthetic miR-34a (black, diamond) miR-34b (grey, square) and miR-34c (light grey, triangle). Graphed is the average of 2 independent experiments run in triplicate. Best fit line was drawn by linear regression modeling. miR-34a/b/c was analyzed in total RNA extracted from cells in log growth phase. Plotted is the average Ct value from 1ng of total RNA; *n* = 2 independent RT-qPCR assays (each run in triplicate), from 2 independent RNA extractions.

Supplementary Fig. 2

```
 psi-miR-34 WT  CGACCGUCACAG-AAUCGACCAACAGAGCU
                  |||||||||| ||||||||||||
   hsa-miR-34a    UGGCAGUGUC UUAGCUGGUUGU
   hsa-miR-34b    AGGCAGUGUCAUUAGCUGAUUGU
   hsa-miR-34c    AGGCAGUGUAGUUAGCUGAUUGC
                  |    ||||  ||||||| |||
 psi-miR-34 MT  CGAGGCACACAC-UAUCGACCAACAGAGCU
```

```
  psi-let-7 WT  CGACUCCAUCAUCCAACAUAUCAAGAGCU
                  ||||||||||||||||||||||
    hsa-let-7a    UGAGGUAGUAGGUUGUAUAGUU
                  |    ||||  |||||||||||
  psi-let-7 MT  CGAGAGGAUCAAGCAACAUAUCAAGAGCU
```

```
 psi-miR-17 WT  CGGUUUCACGAAUGUCACGUCCAUCGAGCU
                  |||||||||||||||||||||||
    hsa-miR-17    CAAAGUGCUUACAGUGCAGGUAG
                  |    ||||  ||||||||||||
 psi-miR-17 MT  CGGAAAGACGAUAGUCACGUCCAUCGAGCU
```

Supplementary Figure 2: Schematic diagram of psi-miR reporter target elements. Schematic diagram of the psi-miR WT and MT target elements subcloned into the 3'UTR of Renilla luciferase in the psiCHECK2 dual-luciferase reporter plasmid (Promega) and the predicted binding of human (hsa) miRNAs genes (shown 5' left to 3' right). Red indicates mutations made to the WT target element. Pink indicates non-binding nucleotides

Supplementary Fig. 3a

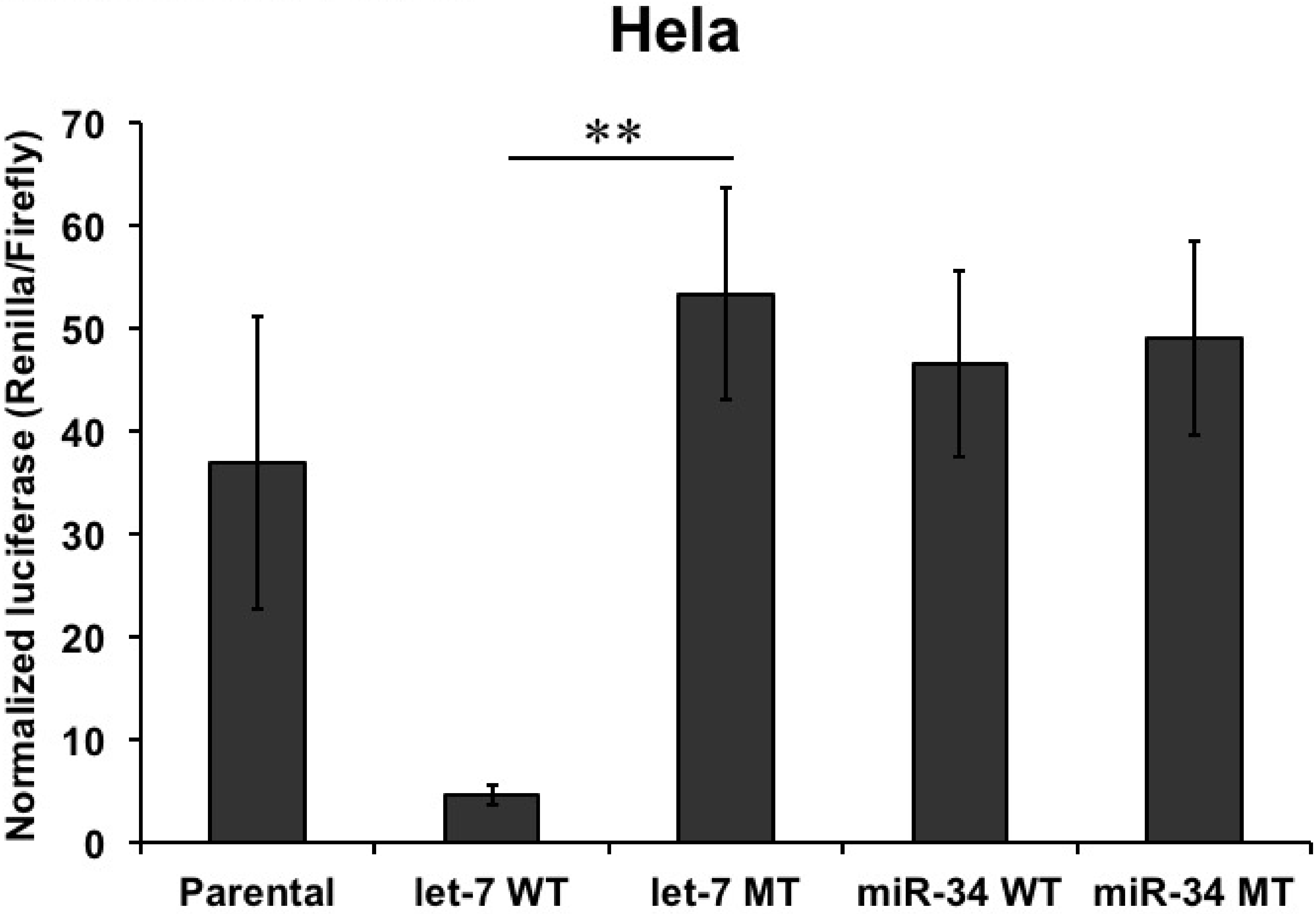

Supplementary Fig. 3b

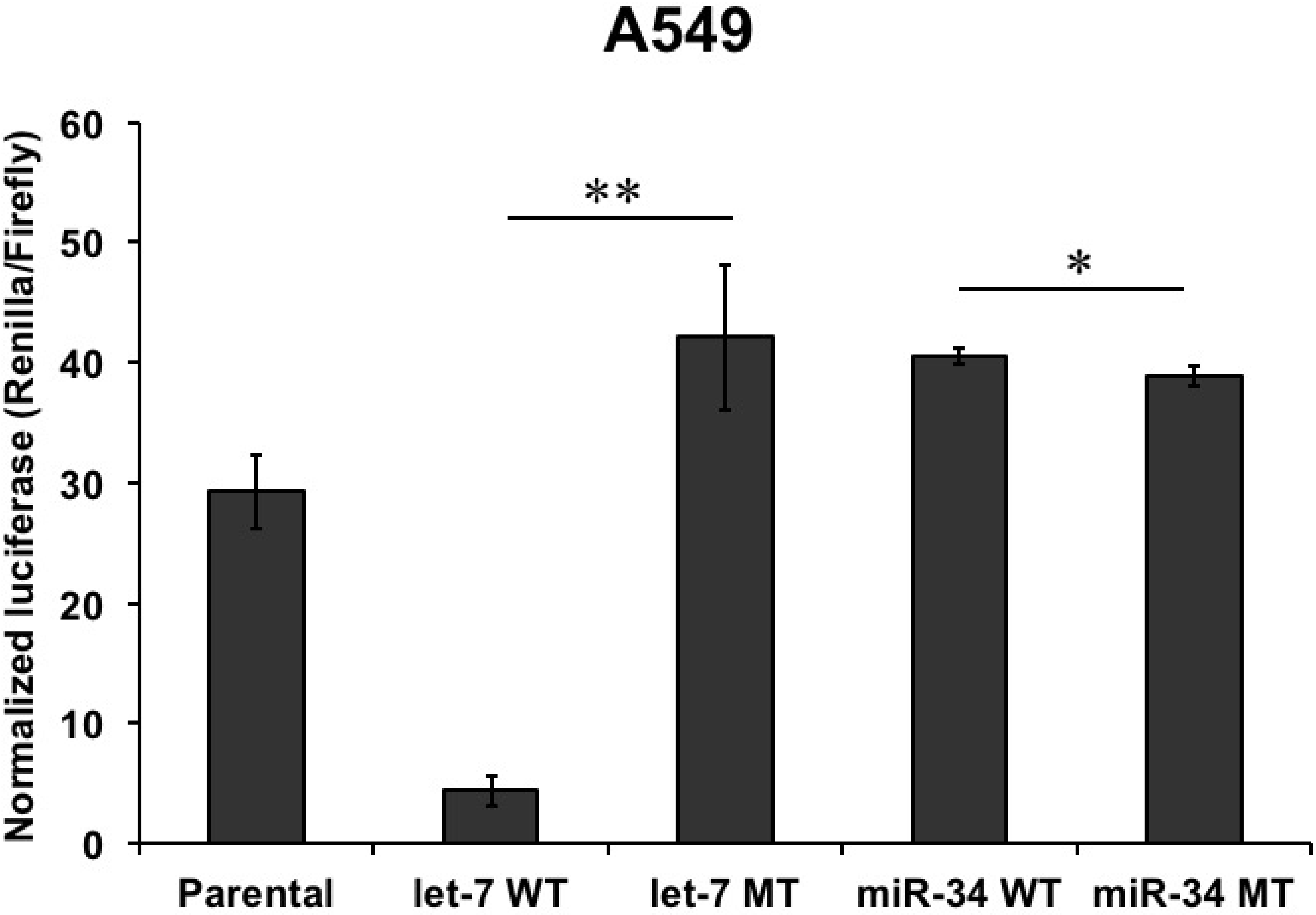

Supplementary Fig. 3c

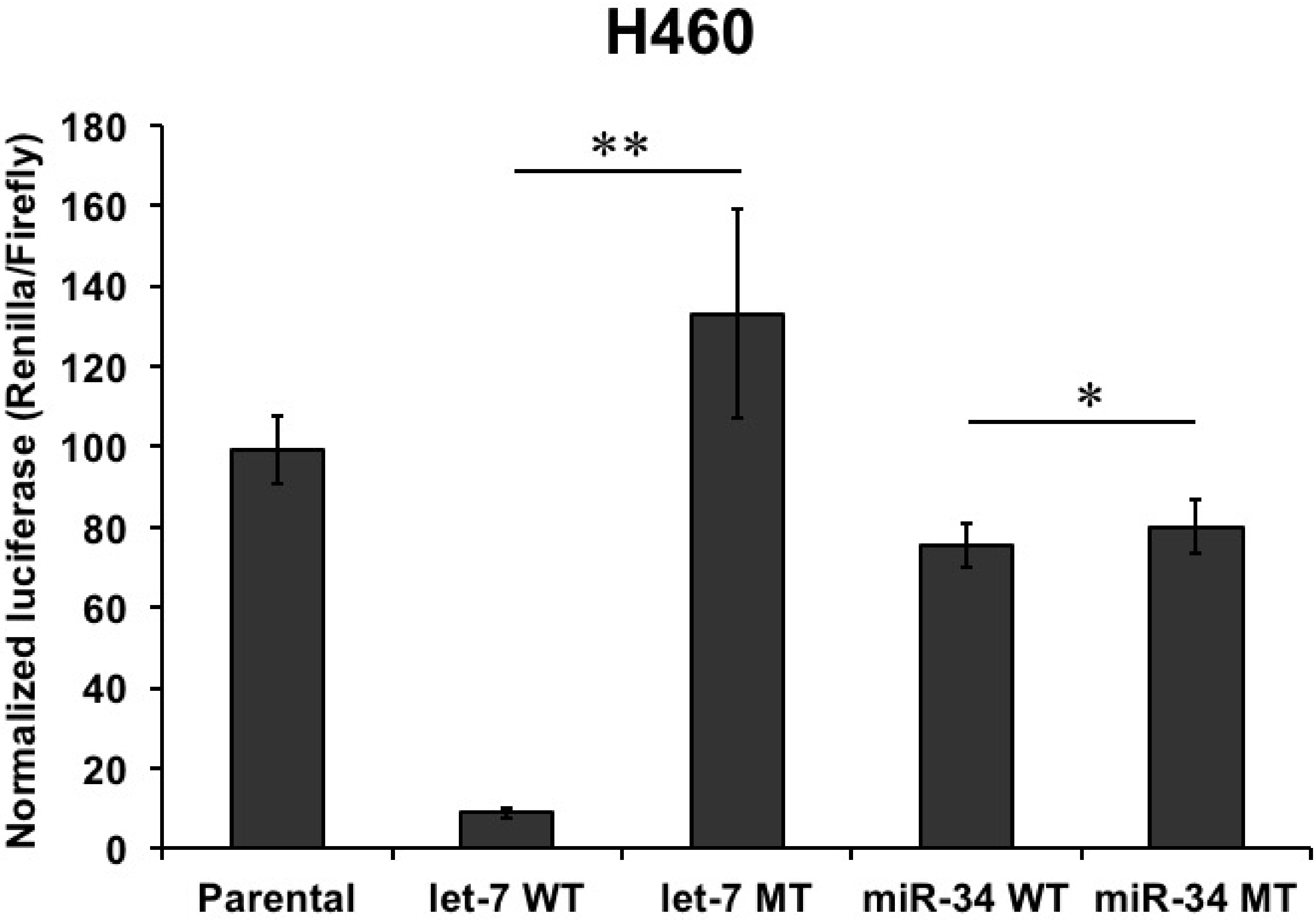

Supplementary Fig. 3d

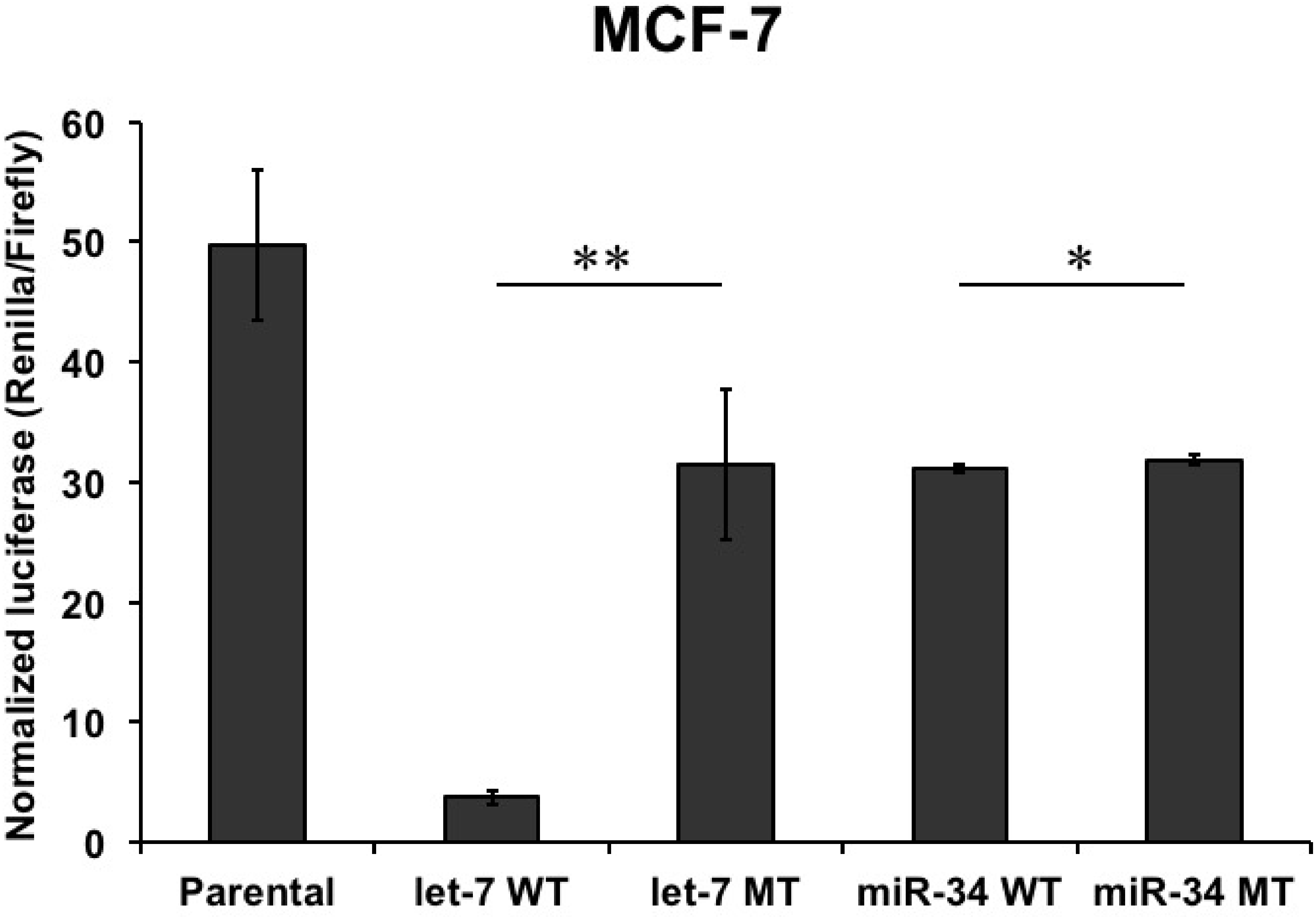

Supplementary Figure 3: Analysis of miR-34 and let-7 activities in various cancer cell lines. Hela (A), A549 (B), H460 (C), MCF-7 (D) cells were transfected with the psiCHECK2, psilet-7 WT, psi-let-7 MT, psi-miR-34 WT or psi-miR-34 MT reporters were analyzed for luciferase activity. Renilla was normalized to Firefly luciferase. Graphed is the average ± s.d. of 3 experiments in triplicate. Asterisk P=0.05, double asterisk P<0.01, one-tailed Student's t-test.

Supplementary Fig. 4

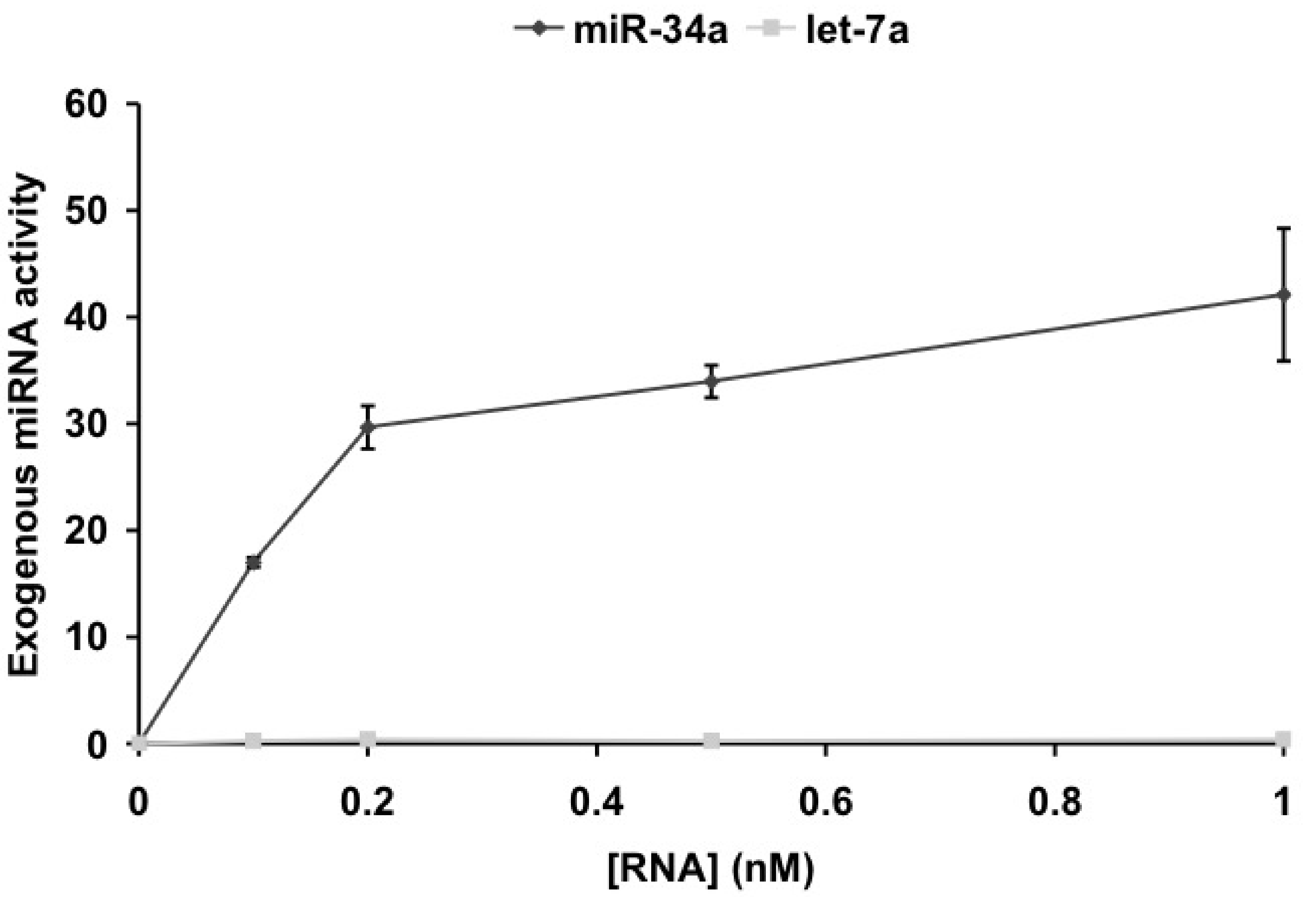

Supplementary Figure 4: Luciferase reporters are responsive to exogenous miR-34. Luciferase activity from A549 cells expressing psi-miR-34 (WT or MT), 16 h post-transfection with either miR-34 or let-7a duplex. Renilla was normalized to Firefly and activity was expressed as the fold- suppression of MT/WT. Data is expressed as the fold change ± s.d., relative to cells not transfected with exogenous miRNA, of 2 experiments in triplicate.

Supplementary Fig. 5

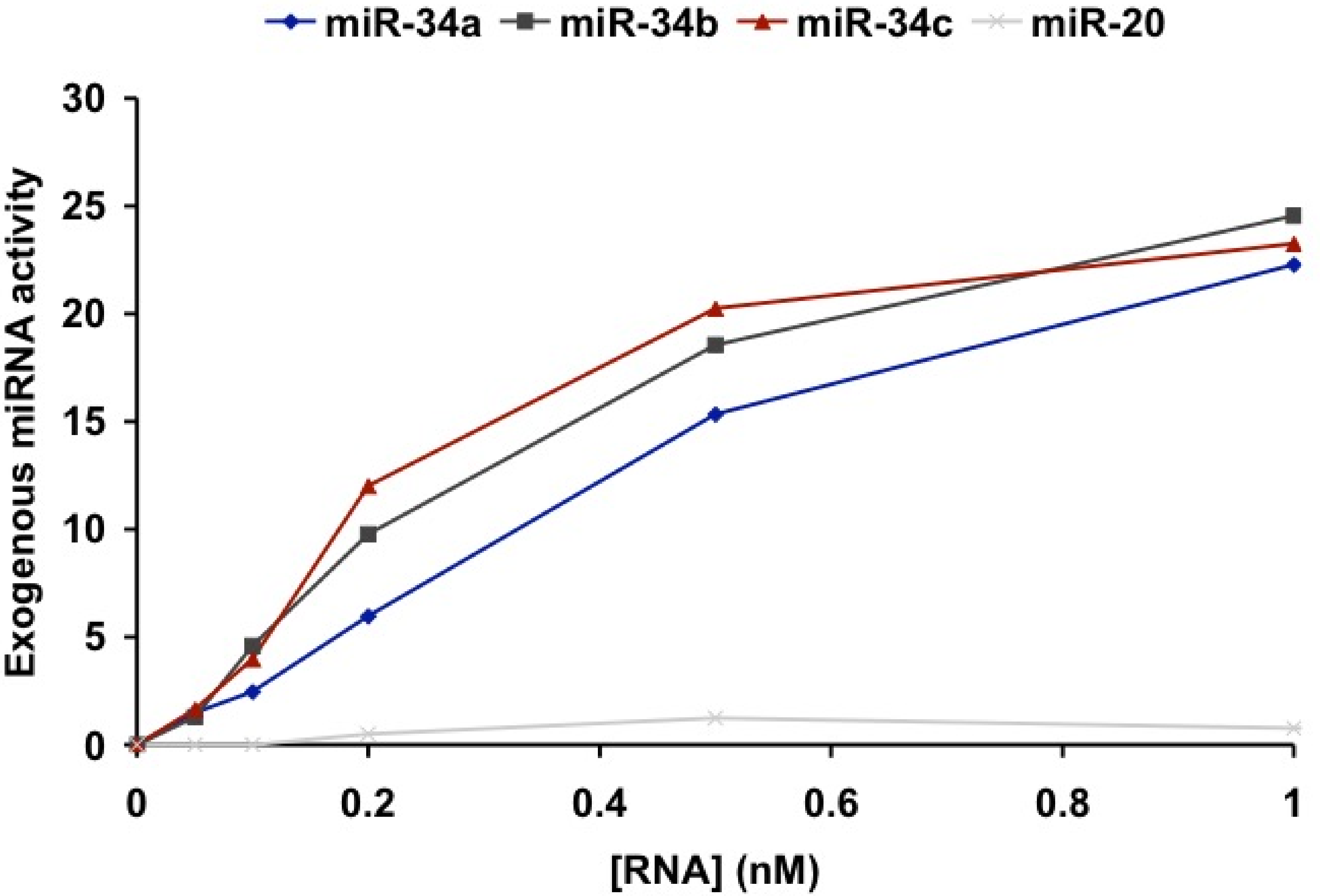

Supplementary Figure 5: psi-miR-34 reporter system measure miR-34a/b/c expression equally. A549 cells expressing the psi-miR-34 (WT or MT) reporter were transfected with synthetic miR-34a, miR-34b, miR-34c or miR-20 RNA duplexes. Luciferase activity was analyzed 16 h post-transfection. Renilla was normalized to Firefly luciferase and the data was expressed as the fold suppression of the MT/WT. Graphed is the average ± s.d. of 2 independent experiments.

Supplementary Fig. 6

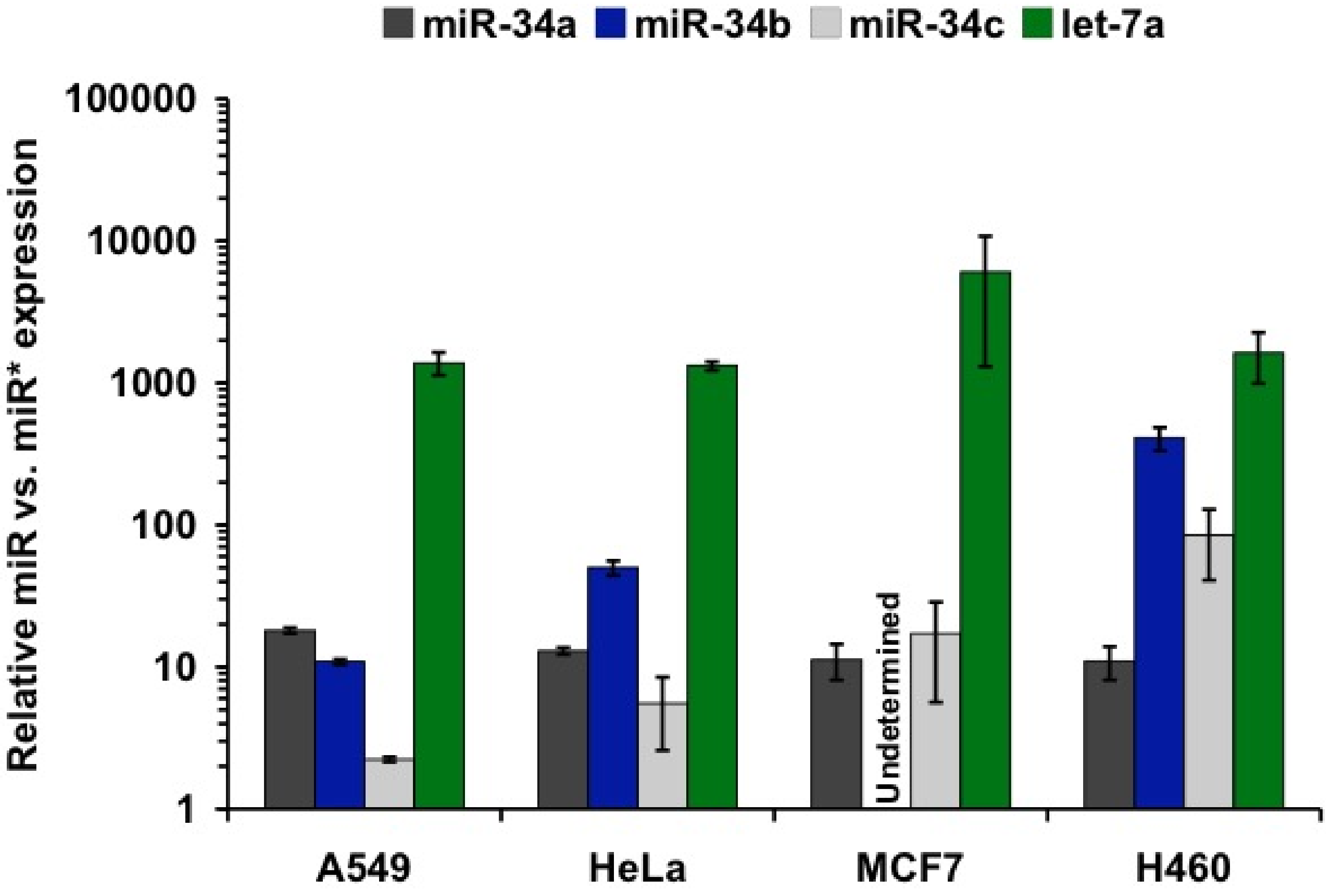

Supplementary Figure 6: Quantification of miR-34 and miR-34* strand in cancer cell lines. RNA extracted from cell lines was analyzed for miR-34a/b/c and miR-34a*/b*/c*. Results were normalized to U6 RNA and quantitated using RT-qPCR. Graphed is the ratio of miR-34 over miR-34*. Undetermined indicates samples where the miR-34* strand was below the limit of detection.

Supplementary Fig. 7

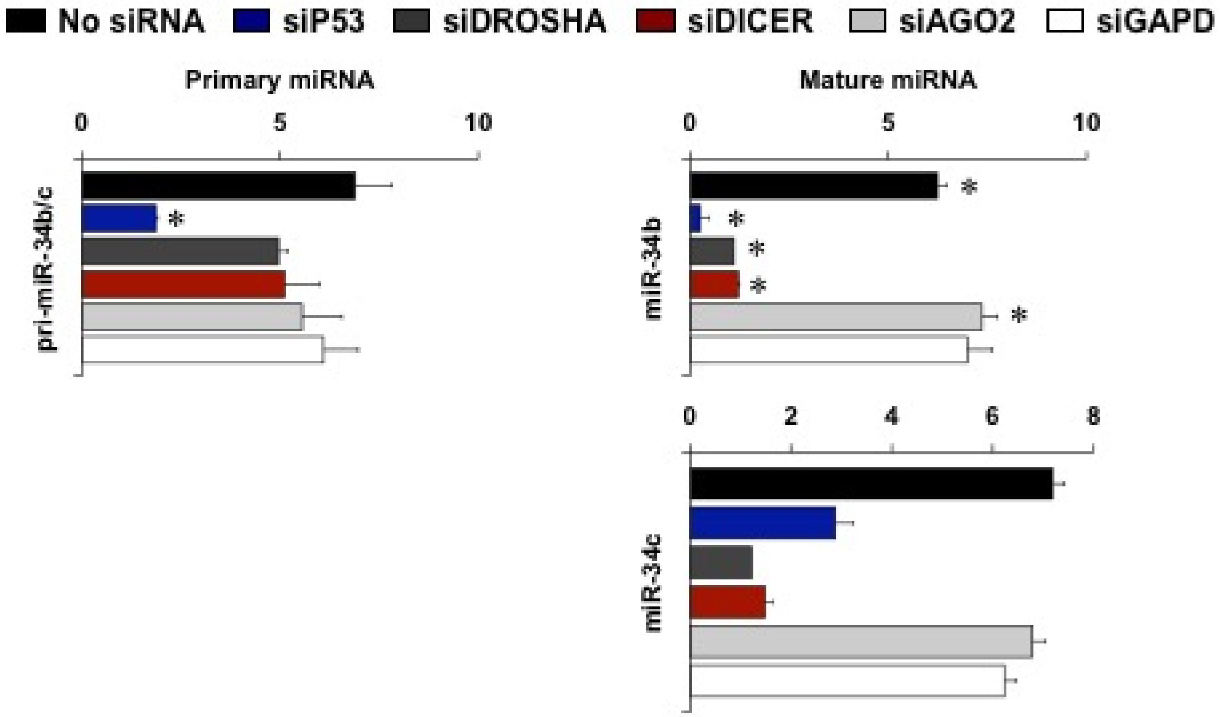

Supplementary Figure 7: Analysis of miR-34b/c expression in siRNA transfected cells. Pri-miR-34b/c, mature miR-34b, and mature miR-34c levels were assayed in cell lysates from **Figure 2b**. Pri-miR-34b/c expression was normalized to ®-Actin mRNA. Mature miR-34b and mature miR-34c expression were normalized to U6 RNA. Graphed is the average ±s.d. of 4 independent experiments. Results are relative to non-irradiated cells.

Supplementary Fig. 8

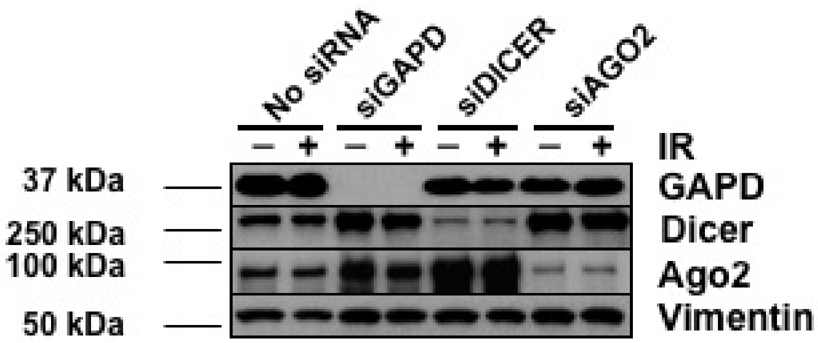

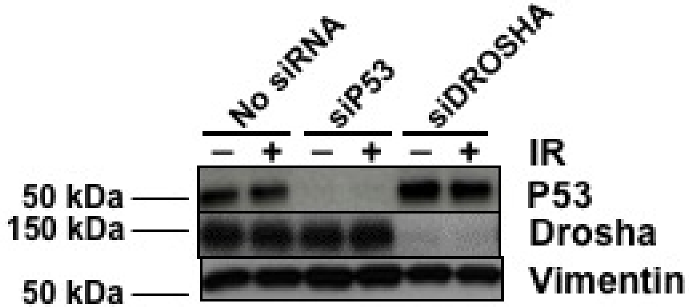

Supplementary Figure 8: Western blot confirms siRNA knockdown. Western blot analysis from lysates of cell transfected with the psi-miR-34 WT reporter, from **Figure 1d**, to confirm protein knockdown at the completion of the experiment.

Supplementary Fig. 9

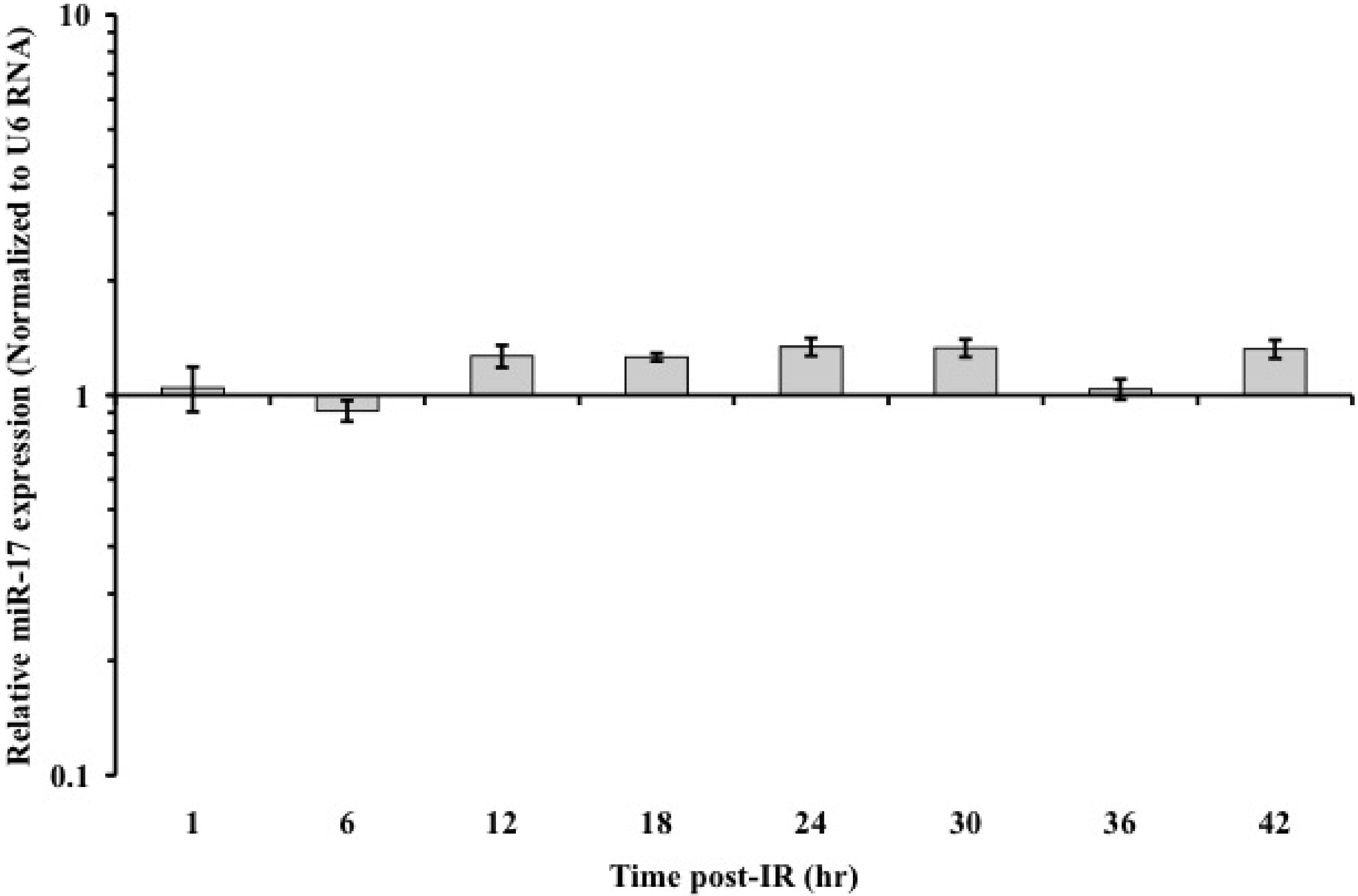

Supplementary Figure 9: miR-17 expression does not change in response to IR. miR-17 expression analyzed from A549 cells exposed to 4Gy of IR and lysed at the indicated time. Graphed is the average and ±s.d. of 2 independent experiments run in triplicate. Results are relative to non-irradiated cells lysed at the same time.

Supplementary Fig. 10

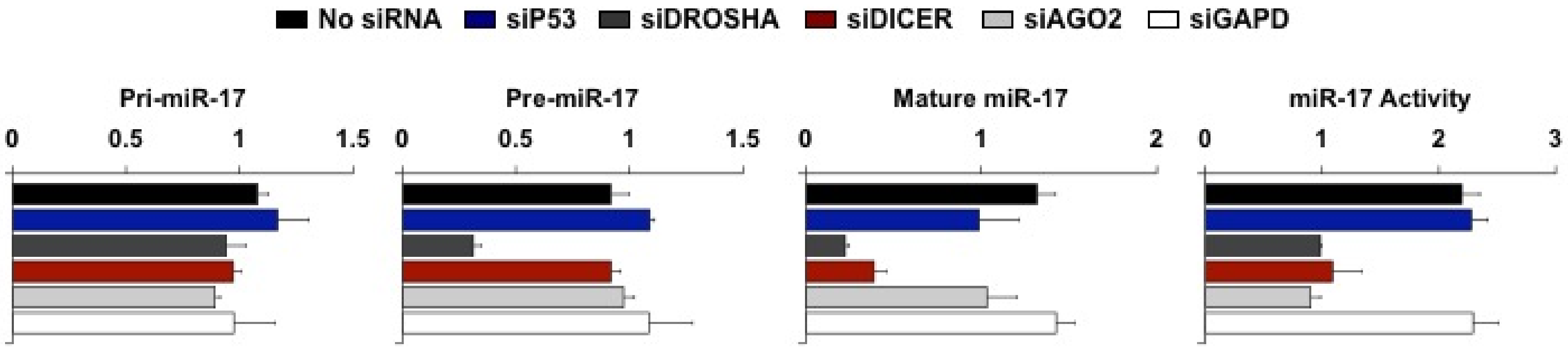

Supplementary Figure 10: Knockdown of miRNA biogenesis genes attenuates miR-17 activity. Cells expressing either the psi-miR-17 WT or psi-miR-17 MT reporter were transfected with siRNA. After a 36 h incubation the cells were lysed and assayed for pri-miR-17, pre-miR-17, mature miR-17 and miR-17 activity. pri-miR-17 and pre-miR-17 were normalized to ®-Actin mRNA. Mature miR-17 was normalized to U6 RNA. Graphed is the average ±s.d. of 4 independent experiments. Results are relative to non-irradiated cells lysed at the same time.

Supplementary Fig. 11

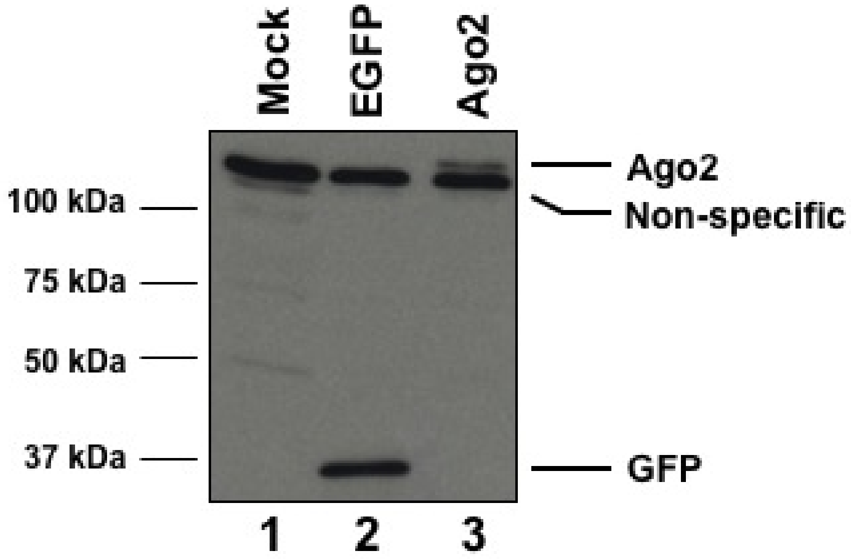

Supplementary Figure 11: Western blot analysis of Flag/HA-tagged EGFP and Ago2. A549 cells transfected with Flag/HA-tagged EGFP or Ago2 were lysed with SDS sample buffer. Lysates were analyzed for protein expression by western blot, probed with anti-HA antibody (HA.C5, Abcam). Mock indicates, lysate from non-transfected A549 cells. Asterisk indicates nonspecific band, used as a loading control.

Supplementary Fig. 12

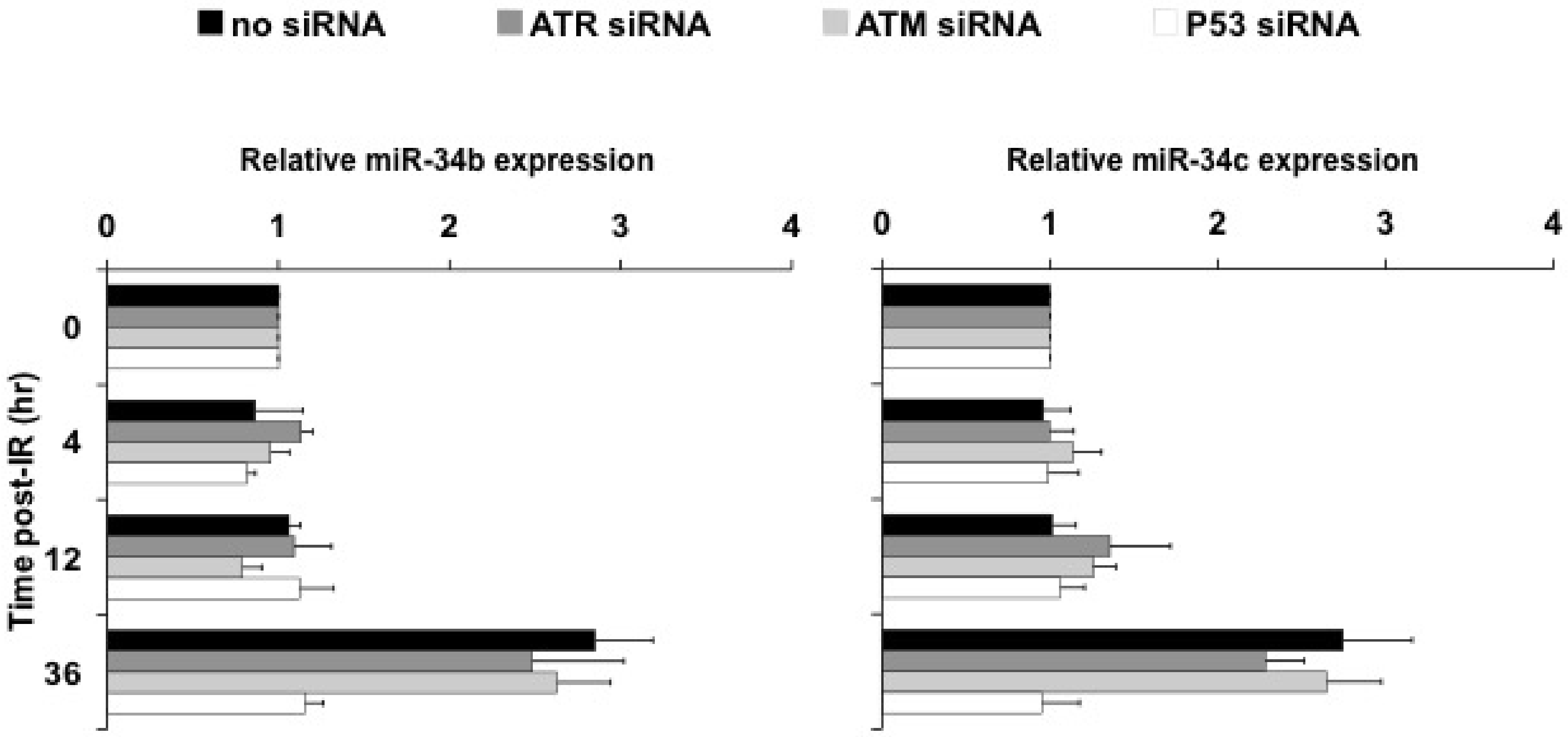

Supplementary Figure 12: Analysis of miR-34b/c expression in ATR, ATM and P53 knockout cell lines. A549 cells expressing the psi-miR-34 (WT or MT) reporters were transfected with siRNA. Irradiated cells (2Gy) were lysed at the indicated time and analyzed for miR-34 expression and activity (**Figure 4a**). Graphed is the average ±s.d. of 4 independent experiments Results are relative to non-irradiated cells, lysed at the 36 h time point.

Supplementary Fig. 13

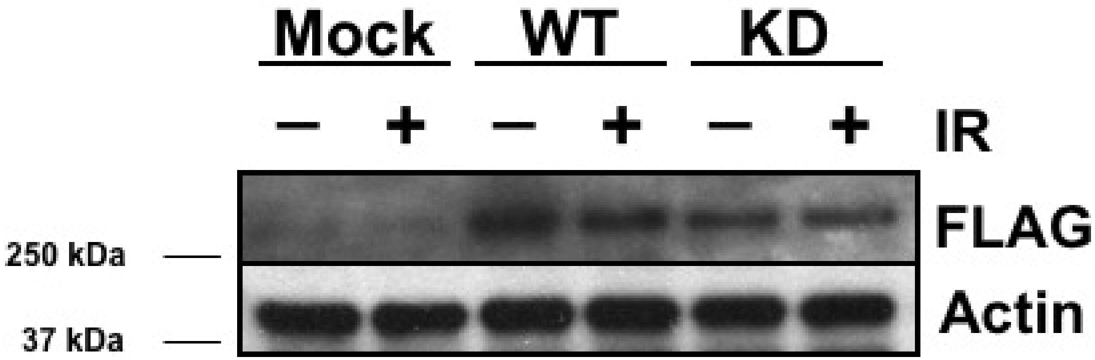

Supplementary Figure 13: Western blot analysis of Flag/His-tagged ATM (WT and KD) in ATM-deficient cells. GM16666 (ATM-deficient) cells were transfected with wild-type (WT) and kinase-dead (KD) Flag/His-tagged ATM. After 24 h, cells were transfected with the psi-miR-34 WT reporter. Cells were lysed 4 h after 2Gy exposure and lysates were analyzed for ATM expression by western blot using anti-Flag antibody. ®-Actin was used as a loading control.

Supplementary Fig. 14

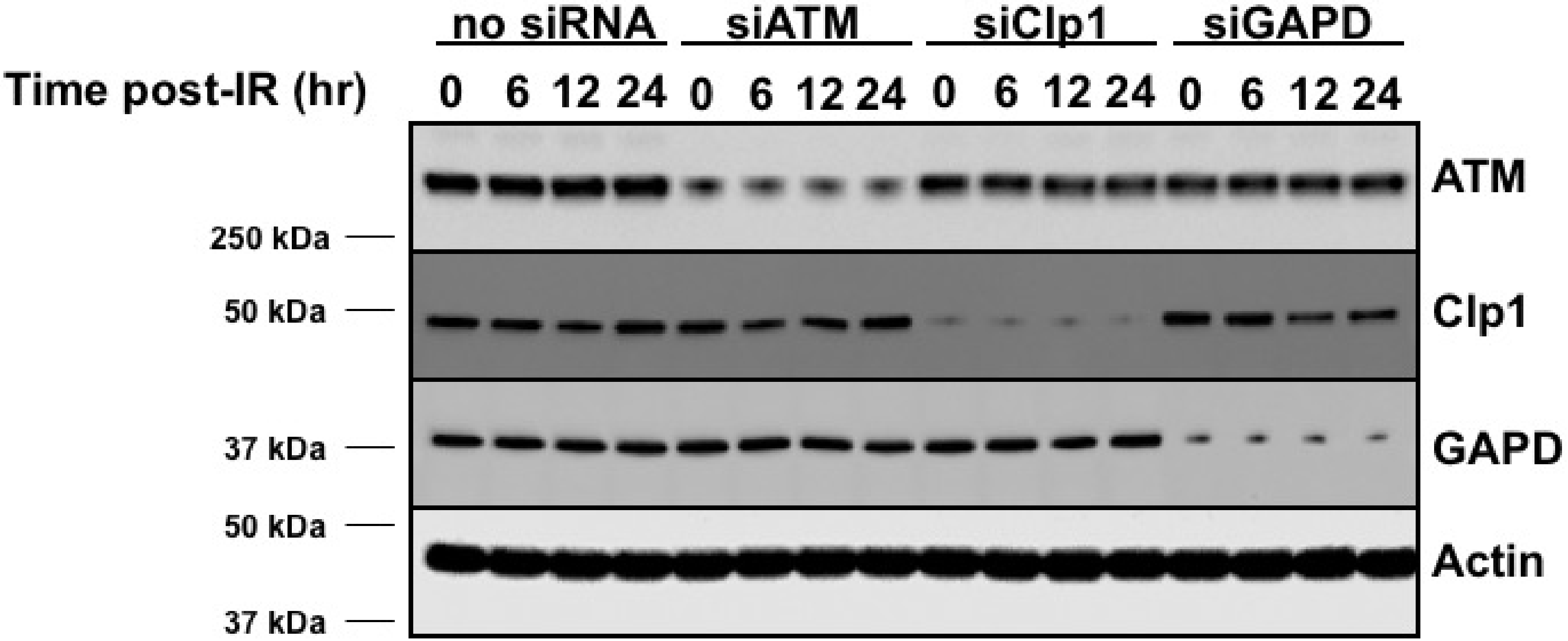

Supplementary Figure 14: Western blot analysis of siRNA knockdown. Western blot analysis from lysates of cell transfected with the psi-miR-34 WT reporter, from **Figure 3b**, to confirm protein knockdown at the completion of the experiment.

Supplementary Fig. 15

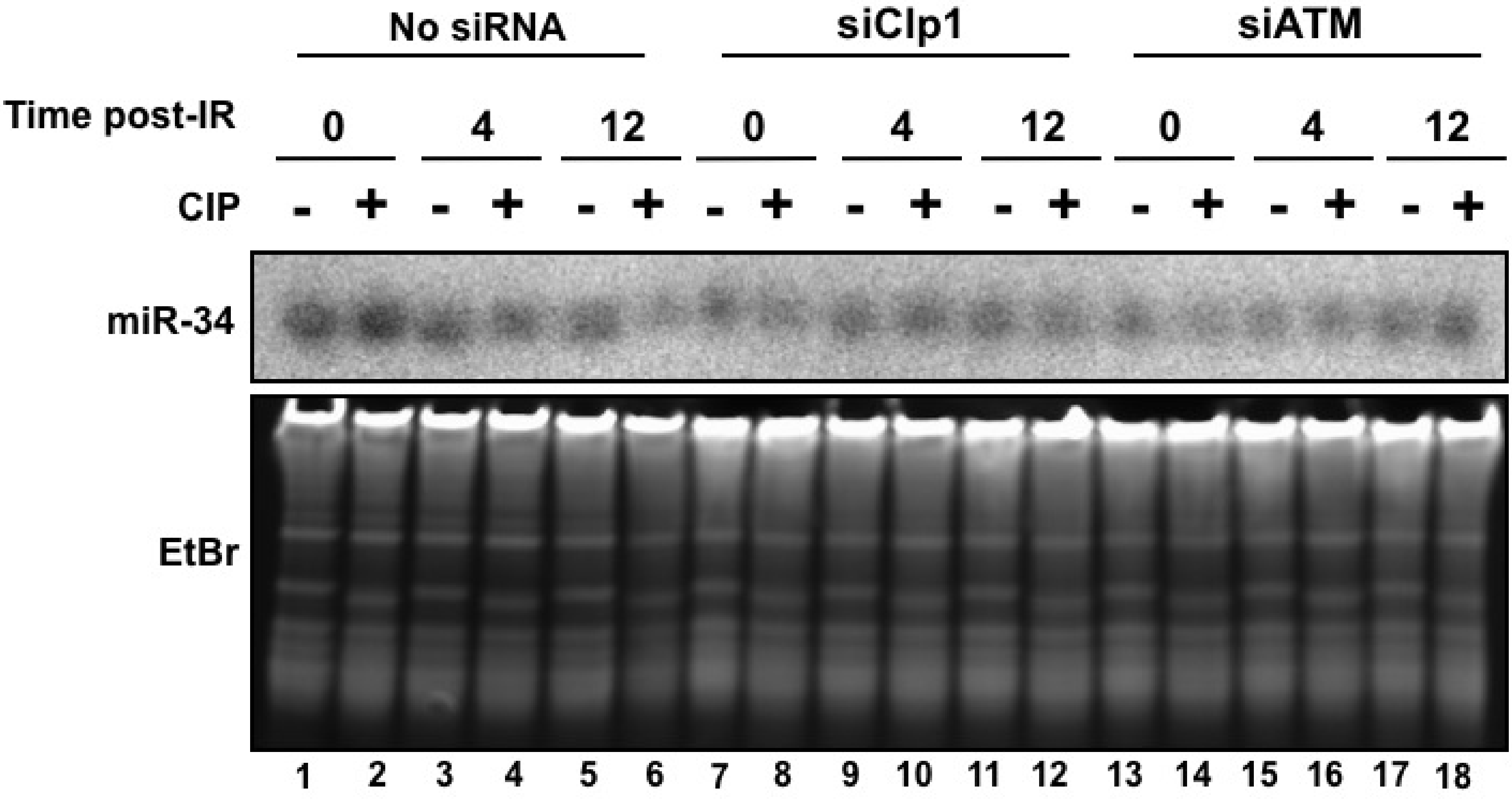

Supplementary Figure 15: hClp1 and ATM are required for IR-induced miR-34 5’-end phosphorylation. miR-34 Northern blot data of CIP-treated total RNA extracted from A549 cells pre-treated with either hClp1 or ATM siRNA and exposed to 2Gy of IR. EtBr staining is used as a loading control.

Supplementary Fig. 16

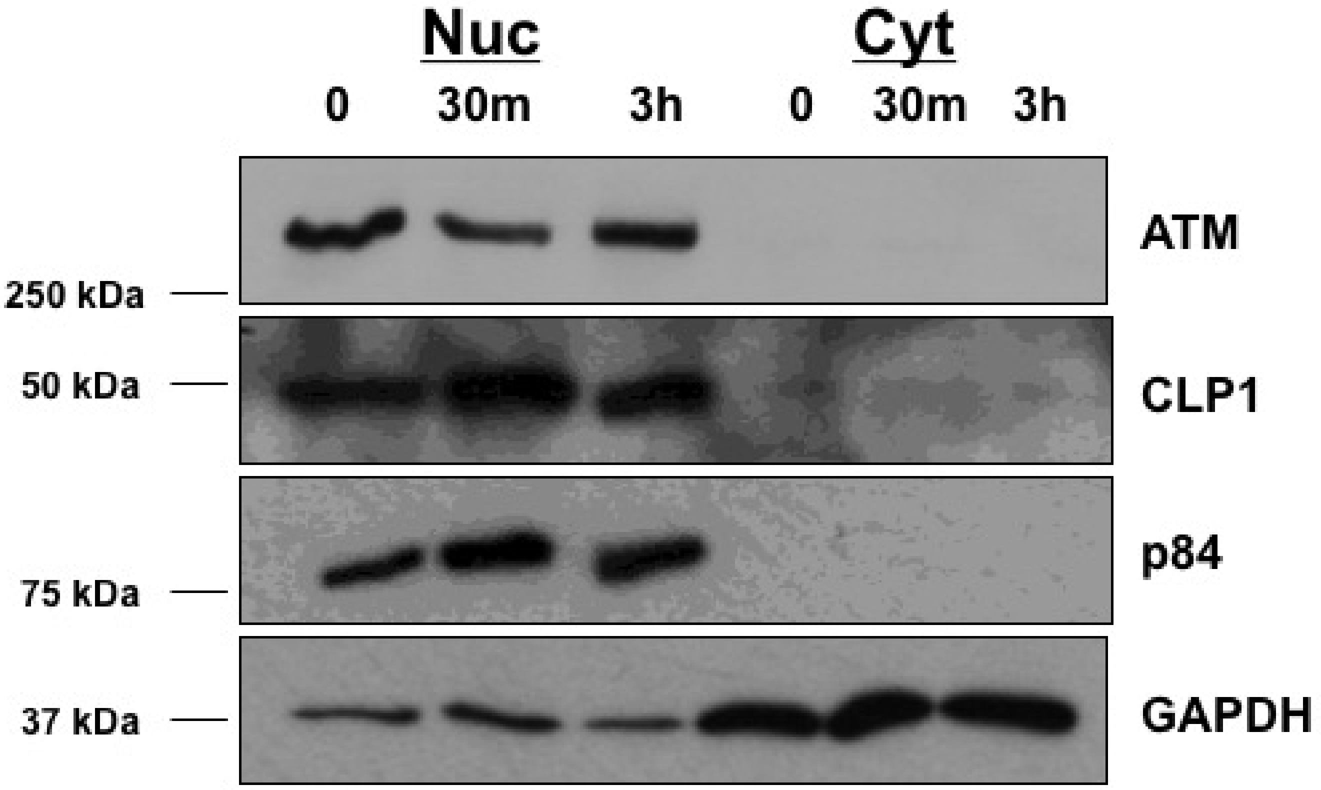

Supplementary Figure 16: Both ATM and CLP1 proteins are expressed primarily in the nucleus. A549 cells were irradiated (4Gy) and nuclear and cytoplasmic proteins were isolated at 0 (no IR), 30 minutes and 3.0 hours after IR**.** 50ug of proteins were loaded in each lane and probed with ATM of CLP1 antibody. p84 (nuclear protein) and GAPDH were used as loading controls to confirm the nuclear and cytoplasmic fractions.

Supplementary Fig. 17

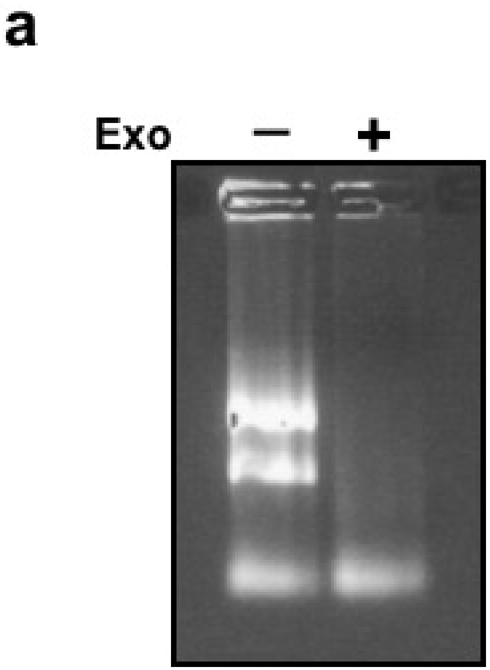

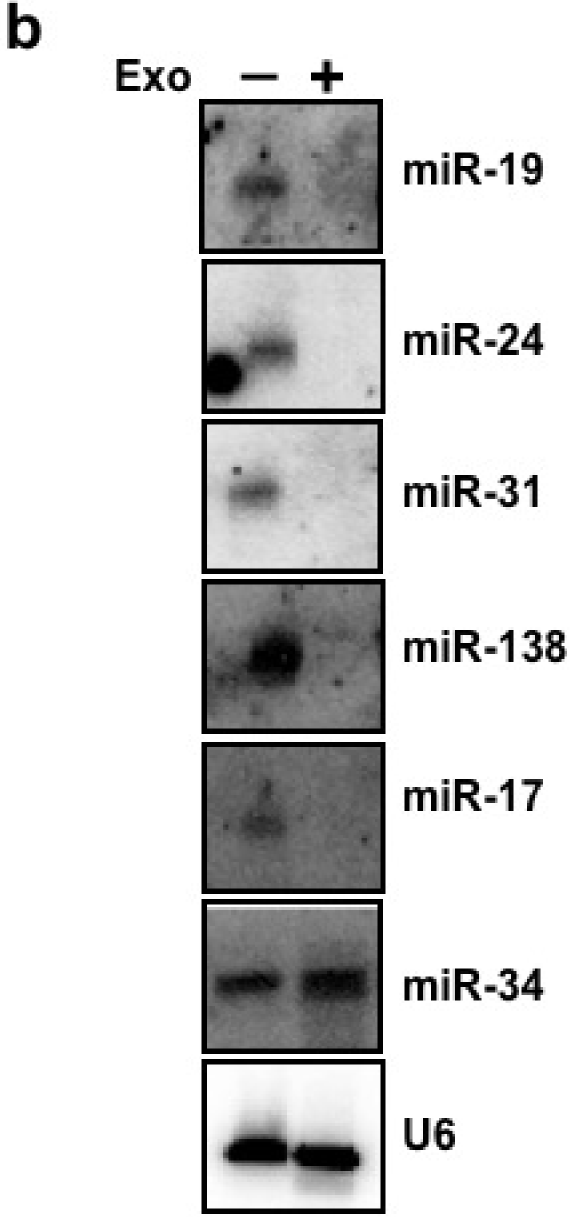

Supplementary Figure 17: 5'-end analysis of miRNAs involved in the radiation response. 50ug of RNA extracted from A549 cells was treated or untreated with Terminator 5'-Phosphate Dependent Exonuclease (10U). **a,** 500ng of RNA was separated using a 1% agarose gel and visualized by ethidium bromide staining. **b,** 30ug of total RNA was separated under denaturing conditions using a 10% polyacrylamide gel with 50% urea. Northern blotting for the indicated miRNAs was performed following the Bartel Lab's (original) Small RNA northern blot protocol.

Supplementary Fig. 18

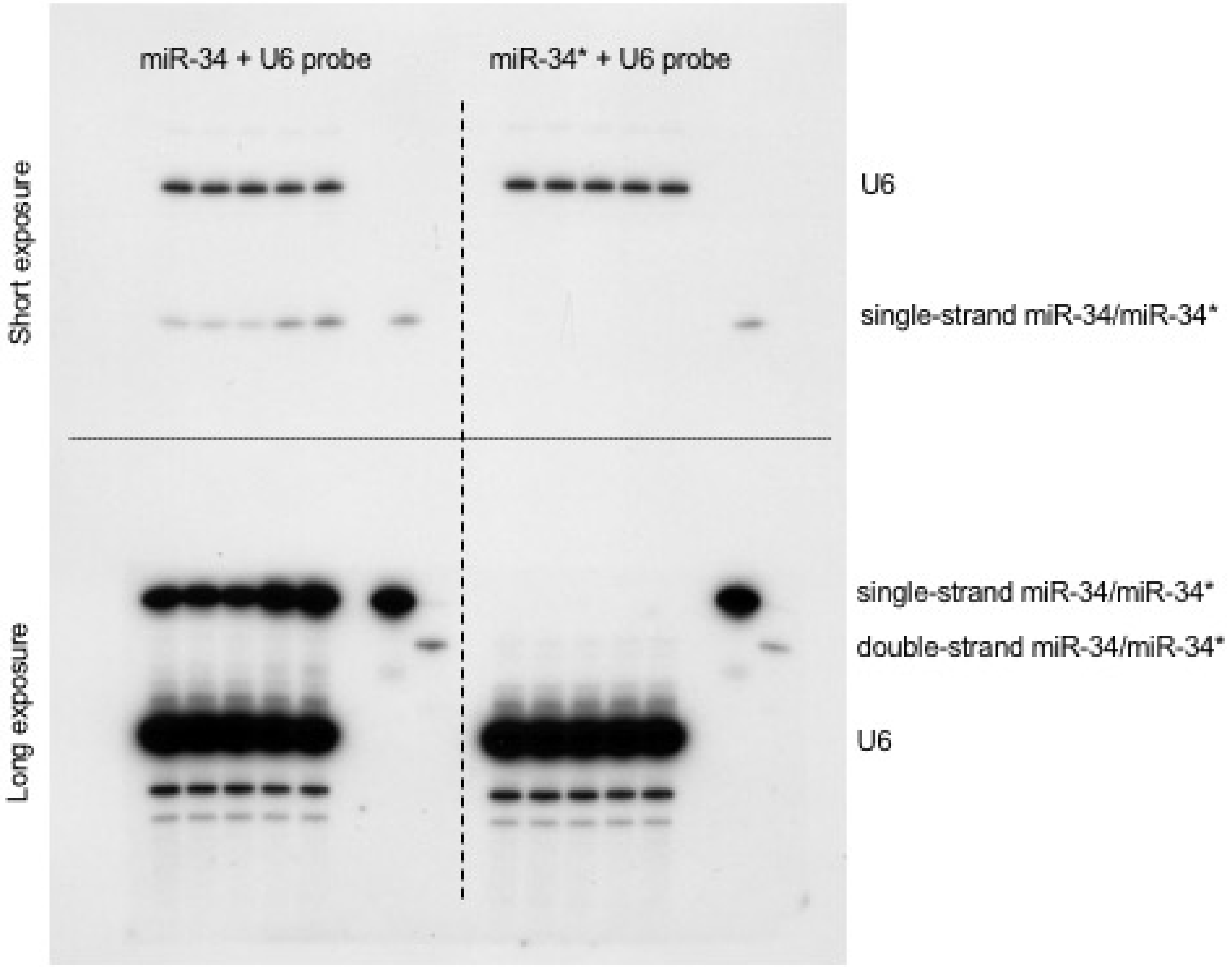

Uncropped scan of Figure 1b
(mirror image short/long term exposures on same film)

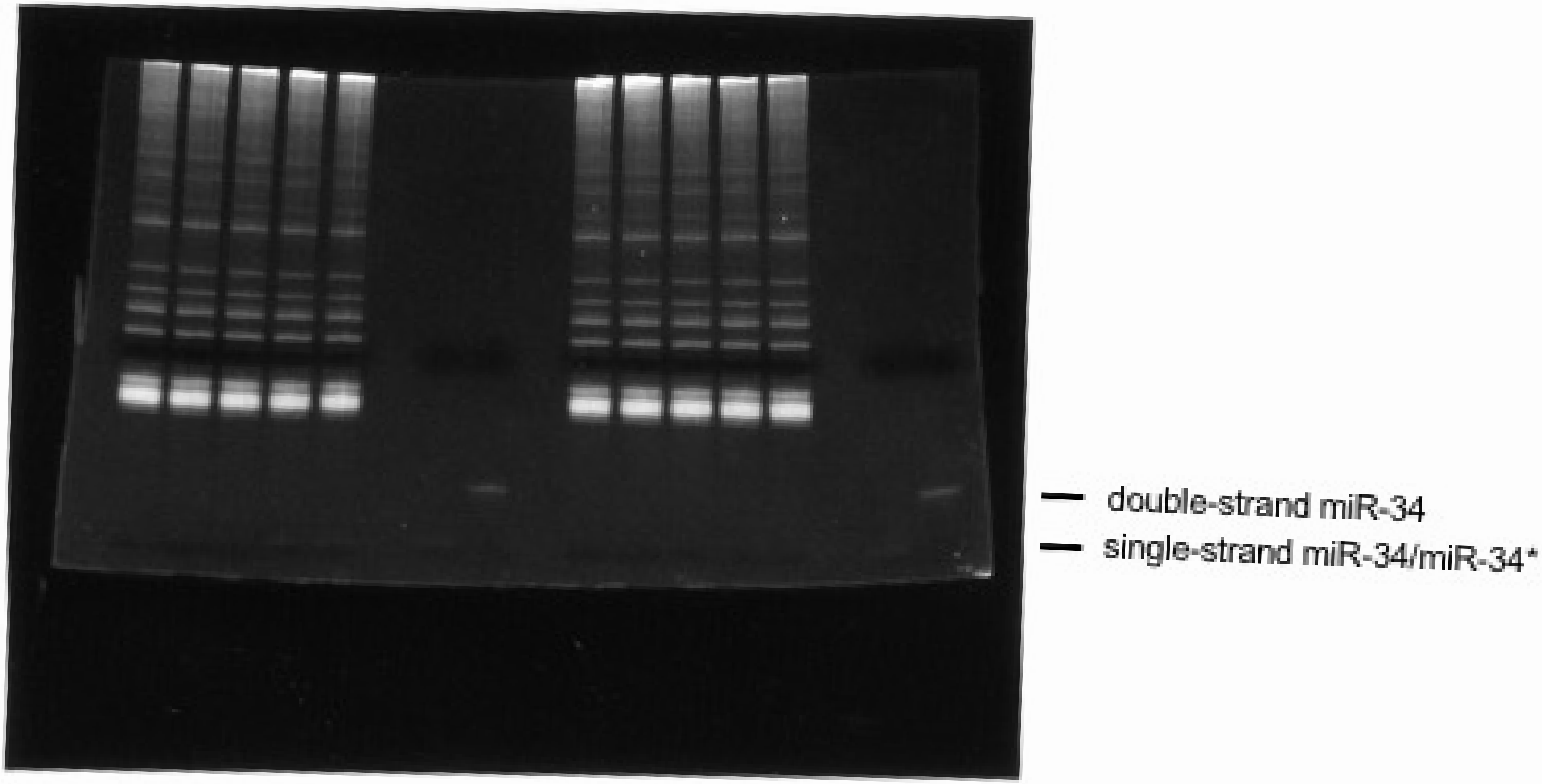

Ethidium Bromide stained gel before transferring RNA to Hybond-N+ membrane

Uncropped scan of Figure 1b

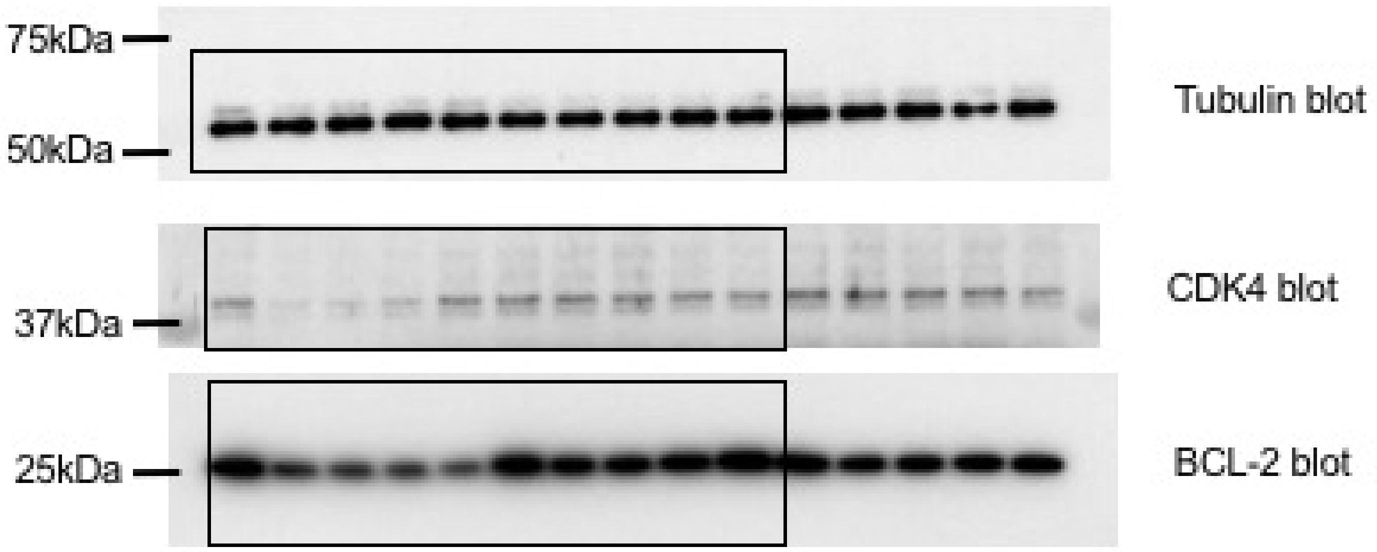

Precision Plus Protein™ Dual Color Standards (Bio-Rad)
Gel: Criterion 4-20% GTX Gradient Gel (Bio-Rad)

Uncropped scans of Figure 1e

Long exposure

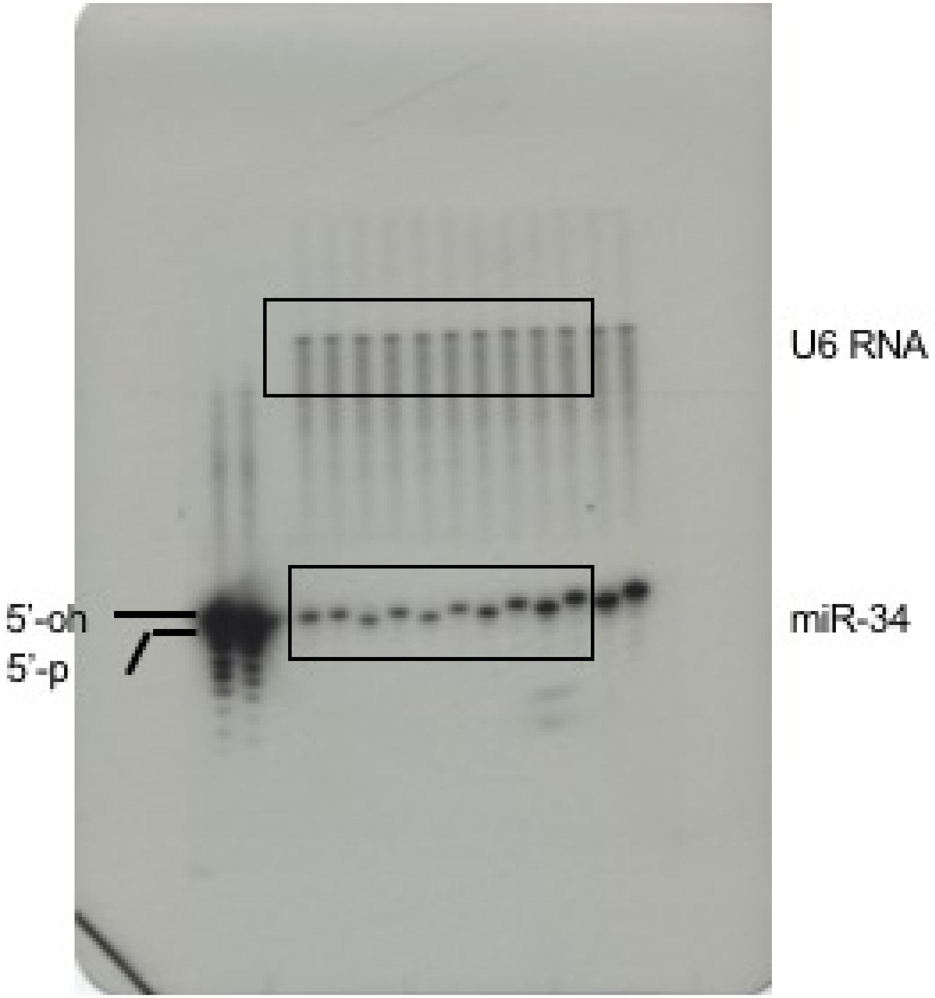

Northern blot of -/+ CIP treated RNA from -/+ IR treated A549 cells using miR-34 and U6 probes

Size markers are synthetic miR-17 bearing a 5'-oh or 5'-p

Uncropped scan of Figure 2b

Short exposure

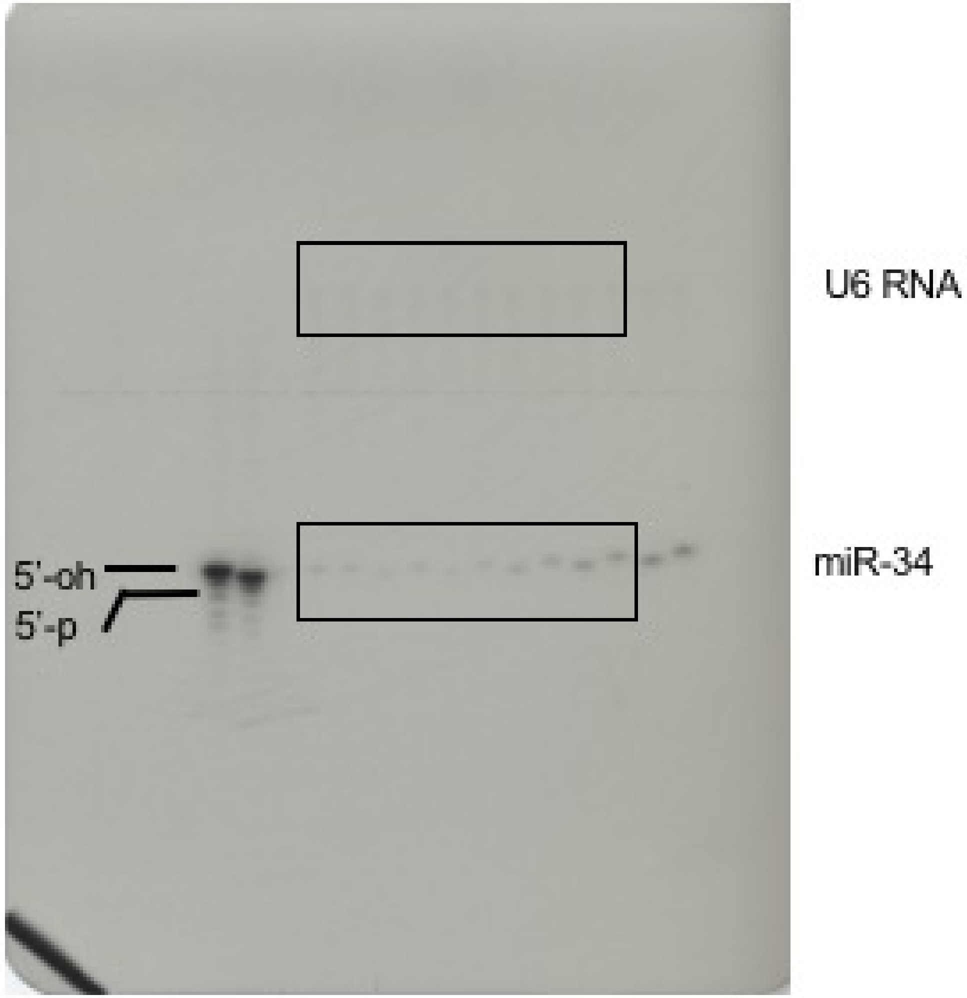

Northern blot of -/+ CIP treated RNA from -/+ IR treated A549 cells using miR-34 and U6 probes

Size markers are synthetic miR-17 bearing a 5'-oh or 5'-p

Uncropped scan of Figure 2b

Long exposure

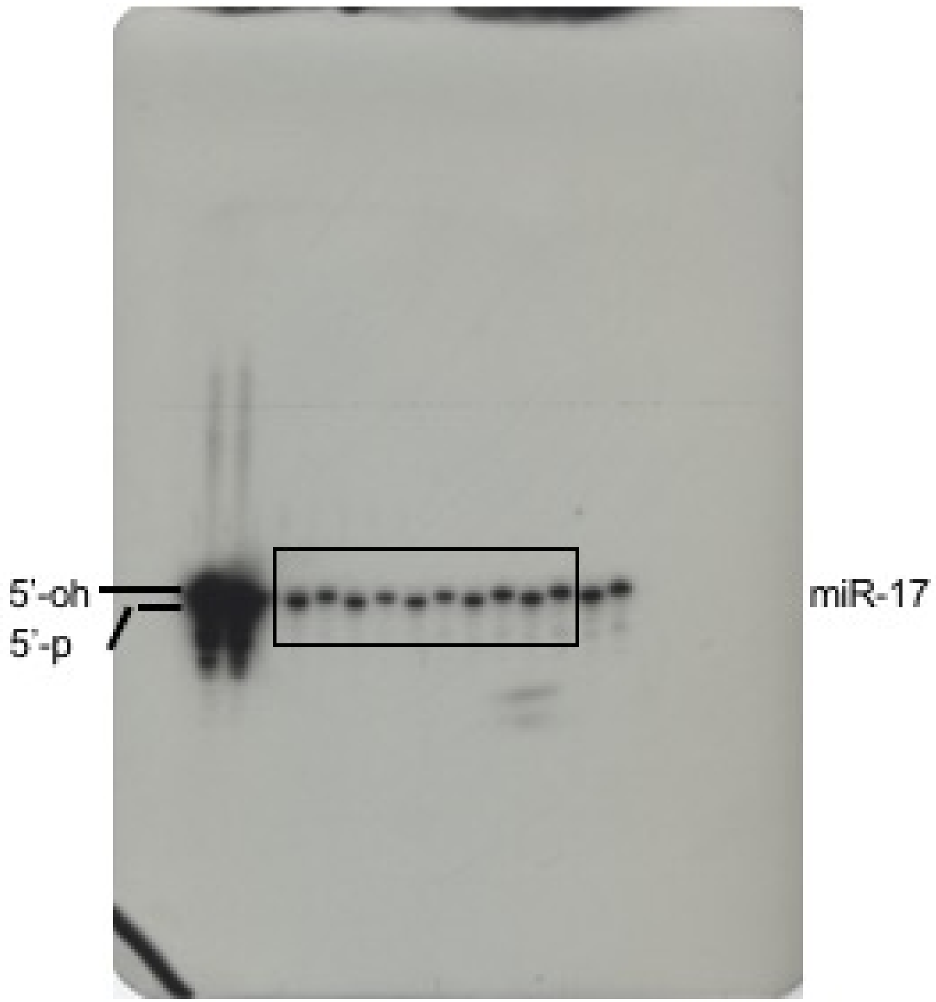

Northern blot of -/+ CIP treated RNA from -/+ IR treated A549 cells using miR-17 probe

Size markers are synthetic miR-17 bearing a 5’-oh or 5’-p

Uncropped scan of Figure 2b

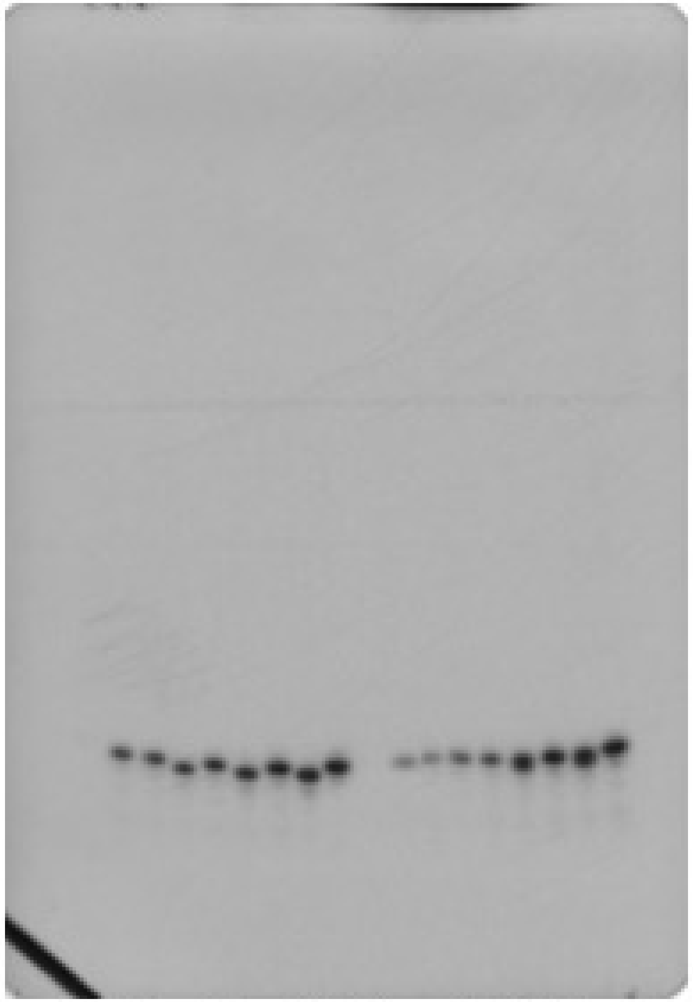

miR-34 northern blot from -/+ CIP treated RNA extracted from A549 cells -/+ IR treated with siControl or siATM

Uncropped scan of Figure 2b

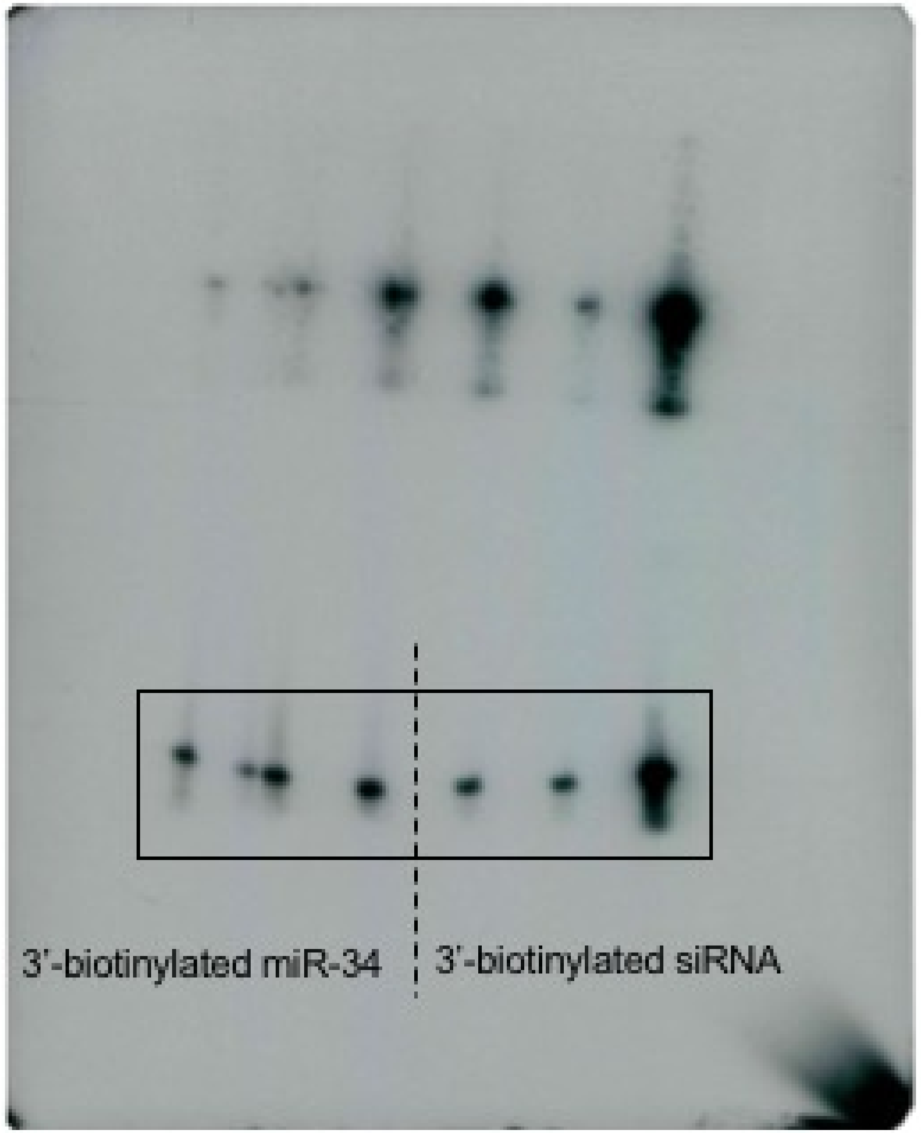

In vitro kinase assay of 3'-biotinylated miR-34 and 3'-biotinylated siRNA incubated with IP'ed proteins

Uncropped scan of Figure 4b

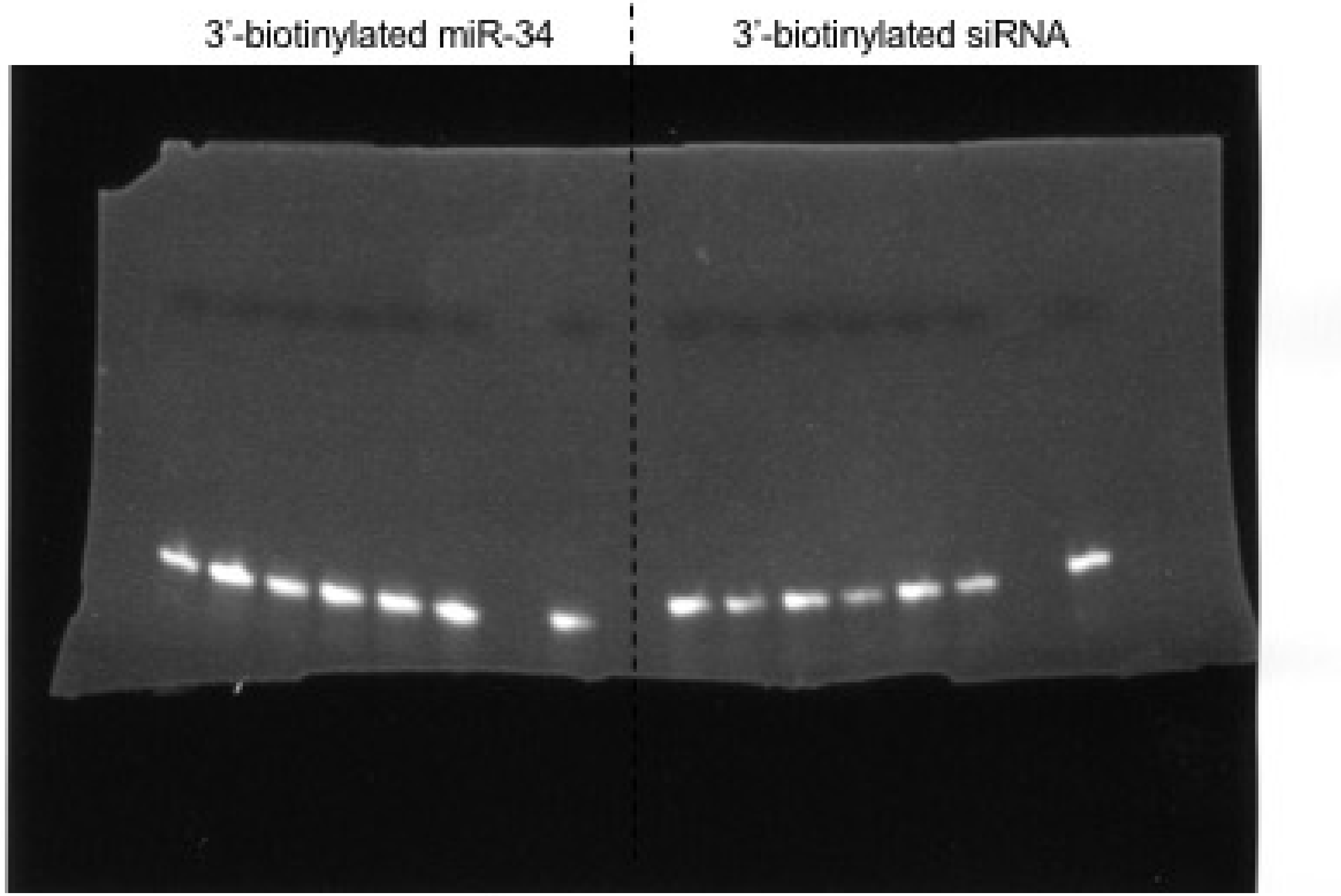

Ethidium bromide stained gel from in vitro kinase assay

Uncropped scan of Figure 4b

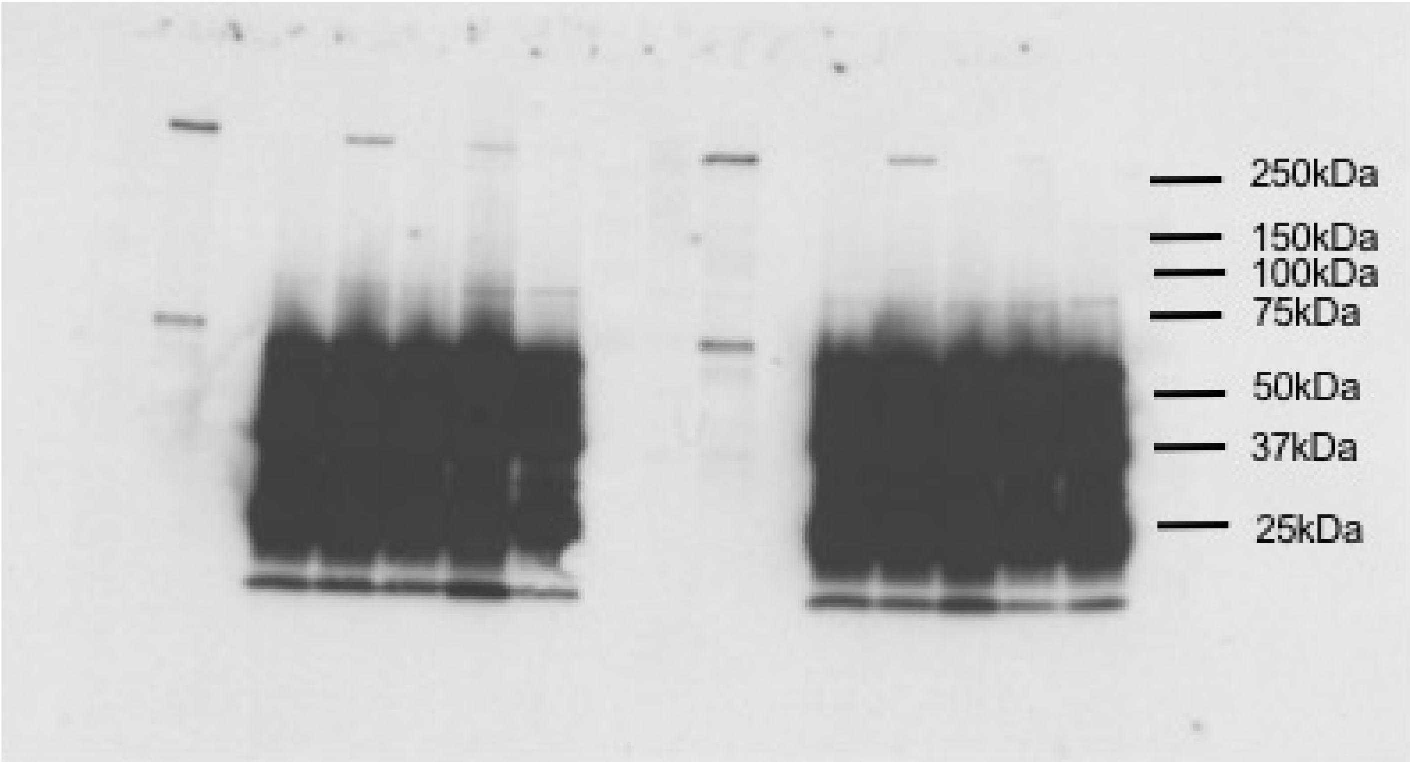

Precision Plus Protein™ Dual Color Standards (Bio-Rad)
Gel: Criterion 4-20% GTX Gradient Gel (Bio-Rad)

Uncropped scans of Figure 4c

Supplementary Tables

Supplementary Table 1: List of oligos used for generating luciferase reporters

Oligo name	Sequence	Length
LET-7 WT F	TCGAGAACTATACAACCTACTACCTCAGC	29
LET-7 WT R	GGCCGCTGAGGTAGTAGGTTGTATAGTTC	29
LET-7 MT F	TCGAGAACTATACAACGAACTAGGAGAGC	29
LET-7 MT R	GGCCGCTCTCCTAGTTCGTTGTATAGTTC	29
MI34 WT F	TCGAGTCAACCAGCTAAGACACTGCCAGC	29
MI34 WT R	GGCCGCTGGCAGTGTCTTAGCTGGTTGTC	29
MI34 MT F	TCGAGTCAACCAGCTATCACACACGGAGC	29
MI34 MT R	GGCCGCTCCGTGTGTGATAGCTGGTTGAC	29
MR17 WT F	TCGAGCTACCTGCACTGTAAGCACTTTGGC	30
MR17 WT R	GGCCGCCAAAGTGCTTACAGTGCAGGTAGC	30
MR17 MT F	TCGAGCTACCTGCACTGATAGCAGAAAGGC	30
MR17 MT R	GGCCGCCTTTCTGCTATCAGTGCAGGTAGC	30

Supplementary Table 2: List of oligos used for northern blots

Target RNA	Northern Blot Probe Sequence 5′-3′
miR-34a-5p	ACAACCAGCTAAGACACTGCCA
miR-19a-3p	TCAGTTTTGCATAGATTTGCACA
miR-24-3p	CTGTTCCTGCTGAACTGAGCCA
miR-31-5p	AGCTATGCCAGCATCTTGCCT
miR-138-5p	CGGCCTGATTCACAACACCAGCT
miR-17-5p	CTACCTGCACTGTAAGCACTTTG
U6	CACGAATTTGCGTGTCATCCTT
